# Longitudinal changes in brain-derived neurotrophic factor (BDNF) but not cytokines contribute to hippocampal recovery in anorexia nervosa above increases in body mass index

**DOI:** 10.1017/S0033291724000394

**Published:** 2024-07

**Authors:** Johanna Louise Keeler, Klaas Bahnsen, Marie-Louis Wronski, Fabio Bernardoni, Friederike Tam, Dominic Arold, Joseph A. King, Theresa Kolb, David M. Poitz, Veit Roessner, Janet Treasure, Hubertus Himmerich, Stefan Ehrlich

**Affiliations:** 1 Centre for Research in Eating and Weight Disorders (CREW), Department of Psychological Medicine, Institute of Psychiatry, Psychology & Neuroscience, King's College London, London, UK; 2 Translational Developmental Neuroscience Section, Division of Psychological and Social Medicine and Developmental Neurosciences, Faculty of Medicine, TU Dresden, Dresden, Germany; 3 Neuroendocrine Unit, Department of Medicine, Massachusetts General Hospital and Harvard Medical School, Boston, MA, USA; 4 Eating Disorder Treatment and Research Center, Department of Child and Adolescent Psychiatry, Faculty of Medicine, TU Dresden, Dresden, Germany; 5 Institute for Clinical Chemistry and Laboratory Medicine, TU Dresden, Dresden, Germany; 6 Department of Child and Adolescent Psychiatry, Faculty of Medicine, TU Dresden, Dresden, Germany

**Keywords:** anorexia nervosa, brain-derived neurotrophic factor, cytokines, hippocampal subfields, hippocampus, structural MRI

## Abstract

**Background:**

Physical sequelae of anorexia nervosa (AN) include a marked reduction in whole brain volume and subcortical structures such as the hippocampus. Previous research has indicated aberrant levels of inflammatory markers and growth factors in AN, which in other populations have been shown to influence hippocampal integrity.

**Methods:**

Here we investigated the influence of concentrations of two pro-inflammatory cytokines (tumor necrosis factor-alpha [TNF-*α*] and interleukin-6 [IL-6]) and brain-derived neurotrophic factor (BDNF) on the whole hippocampal volume, as well as the volumes of three regions (the hippocampal body, head, and tail) and 18 subfields bilaterally. Investigations occurred both cross-sectionally between acutely underweight adolescent/young adult females with AN (acAN; *n* = 82) and people recovered from AN (recAN; *n* = 20), each independently pairwise age-matched with healthy controls (HC), and longitudinally in acAN after partial renourishment (*n* = 58). Hippocampal subfield volumes were quantified using FreeSurfer. Concentrations of molecular factors were analyzed in linear models with hippocampal (subfield) volumes as the dependent variable.

**Results:**

Cross-sectionally, there was no evidence for an association between IL-6, TNF-*α*, or BDNF and between-group differences in hippocampal subfield volumes. Longitudinally, increasing concentrations of BDNF were positively associated with longitudinal increases in bilateral global hippocampal volumes after controlling for age, age^2^, estimated total intracranial volume, and increases in body mass index (BMI).

**Conclusions:**

These findings suggest that increases in BDNF may contribute to global hippocampal recovery over and above increases in BMI during renourishment. Investigations into treatments targeted toward increasing BDNF in AN may be warranted.

## Introduction

Anorexia nervosa (AN) is a psychiatric disorder characterized by fears of food and weight gain, distorted body image, and a drive for thinness, which contributes to a significantly low weight (American Psychiatric Association, 2014). The cognitive profile associated with AN is indicative of aberrant brain function, including difficulties with cognitive flexibility, central coherence, memory, attention, and visuospatial representation (Adoue et al., 2015; Reville, O'Connor, & Frampton, 2016; Roberts, Tchanturia, & Treasure, 2010; Stedal, Broomfield, Hay, Touyz, & Scherer, 2021; Stedal, Frampton, Landrø, & Lask, 2012). In acute AN, global reductions in grey, and to a lesser extent white, matter of the brain have been observed (Seitz, Herpertz-Dahlmann, & Konrad, 2016; Walton et al., 2022; Wronski et al., 2022), and recent methodological advances have enabled the identification of regionally specific changes in subcortical areas (e.g. the amygdala; Wronski et al., 2022). Undernutrition may particularly affect the structure and function of parts of the brain associated with a high metabolic rate (Bahnsen et al., 2022) such as the hippocampus, where neurogenesis persists throughout the lifespan (Toda, Parylak, Linker, & Gage, 2019).

The hippocampus is a small subcortical structure within the limbic system, in the medial temporal lobes of both hemispheres, consisting of 12 functionally and structurally distinct subfields (Sämann et al., 2022). Its functions have been divided into dorsal and ventral streams (Fanselow & Dong, 2010), the former associated with memory and learning, and the latter largely associated with mood, emotion, and stress responses (Fanselow & Dong, 2010). Structural and functional alterations in the hippocampus have been observed across many psychiatric disorders including affective (Sheline, 2011) and anxiety disorders (Ahmed-Leitao et al., 2019), and are a hallmark feature of Alzheimer's disease (Fjell, McEvoy, Holland, Dale, & Walhovd, 2014). Several studies have shown reduced hippocampal volumes in individuals with AN in the acute underweight state, which may partially normalize following weight restoration, although few longitudinal studies exist (Keeler et al., 2020). Recent advances in neuroimaging technology allow closer inspection of subfields of the hippocampus, although only three studies thus far have used this approach in AN, finding relatively global hippocampal volume reductions in acute AN (Burkert, Koschutnig, Ebner, & Freidl, 2015; Collantoni et al., 2021; Myrvang et al., 2018).

In addition to changes in brain structure, AN is associated with alterations in endocrinological, immunological, metabolic, and growth factors (Dalton et al., 2018; Duriez, Ramoz, Gorwood, Viltart, & Tolle, 2019; Keeler et al., 2022; Roubalová et al., 2020). For example, meta-analyses have revealed increases in pro-inflammatory cytokines such as tumor necrosis factor (TNF)-*α*, interleukin (IL)-6 (Dalton et al., 2018; Solmi et al., 2015), and IL-1*β* (Solmi et al., 2015) and decreases in growth factors such as brain-derived neurotrophic factor (BDNF) (Brandys, Kas, Van Elburg, Campbell, & Adan, 2011; Keeler et al., 2022). Some of these molecular changes may be reversible with weight restoration; longitudinal decreases in IL-6 (Dalton et al., 2020; Solmi et al., 2015) and increases in BDNF (Keeler et al., 2022) have been observed.

Pro-inflammatory cytokines play a crucial role in the regulation of the immune system by providing signals to coordinate an immune response (Dantzer, 2001). However, chronic increases in pro-inflammatory cytokines can be toxic to neurons and glial cells, contributing to neurodegeneration and the pathogenesis of neurodegenerative, and potentially also psychiatric disorders (Hong, Kim, & Im, 2016). Contrastingly, findings from rodent studies suggest a potential neuroprotective role of pro-inflammatory cytokines such as TNF-*α* by upregulating the expression of BDNF in astrocytes (Saha, Liu, & Pahan, 2006). BDNF is a neuroprotective molecule that plays a role in the growth, differentiation, and survival of neurons (Dechant & Neumann, 2003), particularly in the hippocampus.

There has been a call for neuroimaging research to investigate the contribution of growth factors to brain changes in AN (King, Frank, Thompson, & Ehrlich, 2018), and additionally, the potential role of neuroinflammation remains unexplored. Given that neuroinflammation may be present in AN (Dalton et al., 2018; Solmi et al., 2015) and hippocampal subfields are histologically, cytoarchitecturally and functionally heterogeneous, it may be the case that pro-inflammatory cytokines such as IL-6 and TNF-*α* have subfield-specific effects. For example, one study in people with major depressive disorder has found a negative association between IL-6 and several specific subfields (e.g. the left and right subiculum, and the right granule layer of the dentate gyrus, and cornu ammonis [CA] 1, 3, and 4 [Kakeda et al., 2018]).

Here we investigated for the first time the relationship between peripheral concentrations of two pro-inflammatory cytokines (IL-6 and TNF-*α*) and the neurotrophin BDNF, and volumes of the hippocampus and its subfields, cross-sectionally between adolescent/young adult underweight AN (acAN) and healthy controls (HC) and between individuals long-term recovered from AN (recAN) and HC, as well as longitudinally (i.e. after weight-restoration treatment). Firstly, cross-sectional differences in hippocampal (sub-)regions were examined. We then elucidated whether the aforementioned molecules contributed to any between-group differences whilst controlling for potentially confounding variables (e.g. intracranial volume, age). We hypothesized that increased concentrations of IL-6 and TNF-*α* and decreased concentrations of BDNF in AN would contribute to cross-sectional differences in hippocampal regions between AN and HC. Longitudinally, we investigated whether these molecules contributed to changes in hippocampal (sub-)regions before and after partial weight restoration, over and above increases in weight, hypothesizing that increases in BDNF and decreases in IL-6 and TNF-*α* would contribute to longitudinal increases in hippocampal regions in AN.

## Methods and materials

### Participants

In total, 207 participants participated in the study: 82 underweight patients with acAN (12–28 years old), 20 recAN individuals (17–29 years old), and 105 HC participants (12–28 years old). All participants were female and identified as White European. As in our previous studies, all acAN were admitted for intensive treatment within specialized eating disorder (ED) programs at the child and adolescent psychiatry and psychosomatic medicine department of a tertiary care university hospital. All patients underwent assessments at timepoint 1 (TP1) within 96 h of beginning nutritional rehabilitation (acAN-TP1). A total of 58 acAN were re-examined at timepoint 2 (TP2) after partial weight restoration (>12% increase in body mass index [BMI]; acAN-TP2; M ± s.d. = 88 ± 27 days). Current and past diagnoses of EDs and other pertinent information including potential confounding variables (e.g. medication, comorbidities, menstrual cycle) were obtained in all participants using the expert form of the Structured Interview for Anorexia and Bulimia Nervosa (SIAB-EX) (Fichter, Herpertz, Quadflieg, & Herpertz-Dahlmann, 1998), supplemented with a bespoke semi-structured interview (online Supplementary Materials [SM] 1.2.), and the Mini International Neuropsychiatric Interview (Sheehan et al., 1998) was used to screen for psychiatric comorbidities in HC. Interviews were adapted to DSM-5 criteria (American Psychiatric Association, 2014) and carried out by clinically experienced and trained research assistants. A diagnosis of AN required a BMI below the 10th age percentile (if <15.5 years old) or below 17.5 kg/m^2^ (if >15.5 years old). The recAN group consisted of individuals who previously met AN criteria but had: maintained a BMI > 18.5 kg/m^2^ (or the 10th age percentile if <18 years); regular menstruation; not engaged in significant restrictive eating behavior (or binging/purging), for at least 6 months prior to study participation (M ± s.d. time since AN episode = 59.9 ± 43.3 months, range 12–158 months). HC participants had to have a normal current and previous BMI (18.5–29 kg/m^2^ or between 10th–95th age percentile if <18 years), regular menstruation, and no history of psychiatric illness and were recruited through advertisement among middle/high school and university students. Additional exclusion criteria applied to all study participants included: a history of bulimia nervosa or binge-eating disorder; consumption of any psychoactive drug within the 4 weeks prior to the study (except for selective serotonin reuptake inhibitors and mirtazapine in acAN and recAN participants); current substance abuse; any history of organic brain syndrome, schizophrenia, substance dependence, bipolar disorder, or psychosis not otherwise specified; current inflammatory, neurologic, or metabolic disorders; chronic illness that could affect appetite, eating behavior, or body weight. Study participants were further excluded if they were pregnant or breast feeding, had anemia, if their IQ was <85, or if they were heavy smokers (>15 cigarettes/day). All protocols received ethical approval by the local Institutional Review Board, and all participants (or their legal guardians) provided written informed consent.

Within the larger magnetic resonance imaging (MRI) sample of 1031 participants (see Wronski et al., 2022), 8% were discarded after quality control (QC; online SM 1.4) (acAN-TP1 = 8%; acAN-TP2 = 12%; recAN = 7%; HC = 8%). From this (post-QC) dataset, all participants without cytokine and BDNF data were excluded (*n* = 824, leaving a final sample of *n* = 207). Following this, HC were age-matched to acAN-TP1 and recAN via optimal pair matching pursuing a minimized sum of absolute pairwise distances in the matched sample (Hansen & Klopfer, 2006). The difference in age means between acAN-TP1 and HC was 0.2 years and 0.5 years between recAN and HC.

### Clinical measures

We assessed ED-specific psychopathology with the Eating Disorder Inventory-2 (EDI-2) (Thiel et al., 1997), depressive symptoms with the Beck Depression Inventory-II (BDI-II) (Hautzinger, Keller, Kühner, & Beck, 2009), and general psychopathology with the Symptom Checklist-90-Revised (SCL-90-R Global Severity Index/GSI) (Franke, 2002). General intelligence (IQ) was estimated with the Wechsler Adult Intelligence Scale (WIE; if age >16 years) or the Wechsler Intelligence Scale for Children (HAWIK; if age ⩽16 years) (Petermann & Petermann, 2008). BMI standard deviation scores (BMI-SDS) were computed to provide an age-corrected index of weight-to-height ratio. Demographic and clinical data were managed using a web-based tool (Research Electronic Data Capture; Harris et al., 2009). Further details are provided in online SM 1.1.

### Blood sample preparation and analysis of cytokines and BDNF

Venous blood samples were collected between 7 and 9 a.m. (for acAN-TP1 within 96 h of admission to treatment). To yield blood serum, samples were left to clot for 30 min at 6–8 °C and then centrifuged (2500 × ***g***, 15 min, 5 °C), aliquoted, and stored at −80 °C until analysis. Serum IL-6 and TNF-*α* concentrations were measured in duplicate with commercially available high-sensitivity enzyme immunoassays (IL-6: IBL International GmbH, Hamburg, Germany; TNF-*α*: R&D Systems Inc., Minneapolis, MN, USA). Participants were excluded from analyses if the coefficient of variation between duplicate measurements exceeded 20% for either IL-6 or TNF-*α* (recAN = 2; HC = 2). Only two IL-6 measurements were below the limit of detection of 0.03 pg/ml and were imputed as 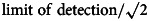. For TNF-*α*, no measurements were below the limit of detection. Free serum BDNF was quantified using commercially available ELISA kits, according to the manufacturer's instructions (R&D Systems, Minneapolis, MN, USA), in three batches. The first batch was measured in duplicate, and the second and third batches were analyzed once. Mean concentrations from the duplicate measurements of the first batch were used in statistical analyses.

### Structural MRI acquisition and image data processing

All participants underwent structural MRI (sMRI) between 8 and 9 a.m. following an overnight fast. High-resolution three-dimensional T1-weighted structural scans were acquired on a Siemens Magnetom Trio 3T Scanner with a magnetization-prepared rapid gradient-echo (MP-RAGE) sequence using the same parameters as in our previous studies (Bahnsen et al., 2022; Bernardoni et al., 2016) (online SM 1.3). Reconstruction of the cerebral cortex was accomplished automatically with FreeSurfer 7.1.1. (Desikan et al., 2006; Fischl et al., 2002), see online SM 1.3. Next, preprocessed images were analyzed with FreeSurfer's automated cross-sectional and longitudinal processing streams (Reuter, Schmansky, Rosas, & Fischl, 2012) followed by the combined amygdala and hippocampus sub-segmentation functionality (Saygin, Osher, Augustinack, Fischl, & Gabrieli, 2011). A standardized QC procedure was performed by trained raters both after reconstruction and after hippocampal sub-segmentation (see online SM 1.4). The following hippocampal (subfield) volumes were examined separately for left and right hemispheres: the whole hippocampus, whole hippocampal body, whole hippocampal head, hippocampal tail, CA1 body, CA1 head, CA3 body, CA3 head, CA4 body, CA4 head, fimbria, granule cell and molecular layer of the dentate gyrus (GC ML DG) body, granule cell and molecular layer of the dentate gyrus head, hippocampus-amygdala transition area (HATA), hippocampal fissure (a cerebral spinal fluid cleft, rather than hippocampal tissue), molecular layer of the hippocampus body, molecular layer of the hippocampus head, parasubiculum, presubiculum body, presubiculum head, subiculum body, and subiculum head. The whole hippocampus encompasses all regions, the whole hippocampal head encompasses all ‘head’ volumes as well as the HATA and the parasubiculum, and the whole hippocampal body encompasses all ‘body’ volumes as well as the fimbria.

### Statistical analyses

Due to deviations from normality, BDNF, IL-6, and TNF-*α* were log10-transformed independently in the cross-sectional and longitudinal samples before parametric tests were applied. As recommended by previous publications (Steinhäuser et al., 2021; Steinhäuser, Wronski, Keeler, Ehrlich, & King, 2022), BDNF values were then adjusted for batch effects and storage time in each sample separately (online SM 1.5). Between-group cross-sectional and longitudinal differences in clinical and demographic characteristics were conducted using *t* tests and analyses of variance. Statistical significance was defined as *p* < 0.05 and Cohen's *d* effect sizes were computed with 0.2, 0.5, and 0.8 representing small, moderate, and large effects, respectively (Cohen, 1992). For all main analyses, we corrected for multiple comparisons using a false discovery rate (FDR) of *q* < 0.05 across all (sub-)regions unless otherwise noted. All statistical analyses were conducted in R Version 4.2.0 (R Core Team, 2022) and an overview of the statistical analysis plan is provided in online SM 1.6.

#### Cross-sectional analyses

For cross-sectional comparisons between acAN-TP1 and HC, and recAN and HC, left and right hippocampi and hippocampal subfield volumes were firstly modeled using general linear models (GLMs) with the study groups as the predictor and age, age^2^, and estimated total intracranial volume (eTIV; correction for head size variation [Sargolzaei et al., 2015]) as covariates. Age was modeled up to the second polynomial order due to evidence for non-linear age effects on hippocampal volumes (Chen et al., 2016; Sämann et al., 2022; Vinke et al., 2018) and in line with recent ENIGMA studies (Han et al., 2021; Zugman et al., 2022).

In a second stage, hippocampal regions that were nominally different between groups (i.e. before FDR correction) were entered into subsequent separate GLMs, investigating the effect of (a) TNF-*α*, (b) IL-6, and (c) BDNF on between-group differences in hippocampal (sub-)regions, using the same aforementioned covariates. Interaction terms between group and cytokines/BDNF are presented in the main manuscript (main effects of molecule in online Supplementary Tables S3 and S4).

#### Longitudinal analyses

Based on previous studies (Bahnsen et al., 2022; Bernardoni et al., 2016), we assumed that the longitudinal differences in grey matter (GM) measures following short-term weight restoration are a linear function of changes in BMI-SDS. However, here we sought to examine whether changes in cytokine/BDNF levels contribute to explain variance in hippocampal changes above and beyond changes in BMI-SDS. Again, only hippocampal regions that were nominally different as a function of increases in BMI-SDS were entered into subsequent linear mixed effects (LME) models investigating the contribution of changes in cytokines/BDNF. In summary, and aligning with prior publications (Bahnsen et al., 2022; Bernardoni et al., 2016), we modeled hippocampal (sub-)region volumes (HV) as:

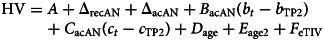
where *b*_*t*_ and *c*_*t*_ represent BMI-SDS and the log-transformed serum levels of TNF-*α*, IL-6, or BDNF, respectively, at timepoint *t*. ΔrecAN and ΔacAN represent group differences in comparison to controls (HV recAN–HV HC; HV acAN-TP2–HV HC, respectively). This approach allowed us to test for the speed of longitudinal changes associated with BMI-SDS, cytokine/BDNF levels (acAN-TP1 *v.* acAN-TP2), and both age and age^2^, which was described in full by Bernardoni et al. (2016).

#### Sensitivity and exploratory analyses

Where cross-sectional or longitudinal associations between TNF-*α*, IL-6, or BDNF and volume (or volume change) of hippocampal (sub-)regions emerged, supplementary models repeated these analyses after (a) removing participants with the AN-BP subtype, and (b) removing participants on antidepressant medication (see online SM 2).

Pearson's correlation coefficients were calculated to investigate possible associations between BDNF and concentrations of IL-6 and TNF-*α* both cross-sectionally and longitudinally (online Supplementary Table S7).

## Results

### Cross-sectional results

#### Demographic and clinical characteristics, cytokines, global GM

Sample characteristics are summarized in Table 1. As expected, acAN-TP1 had a lower current BMI and BMI-SDS and higher levels of psychopathology than HC. AcAN-TP1 had a lower total subcortical GM volume than HC. RecAN had a lower minimum lifetime BMI and higher socioeconomic status, ED psychopathology, and depression, compared with HC. There were no cross-sectional differences in TNF-*α*, IL-6, or BDNF between acAN and HC or recAN and HC and these results did not change when removing participants who smoke or participants with a current/past AN-BP subtype (online SM 2.1.1). When removing participants on antidepressants, TNF-*α* was lower in acAN-TP1 compared with HC, but not different between recAN and HC (online SM 2.1.1).

**Table 1.** Demographic and clinical measures from cross-sectional samples

Variable	Age-matched acAN *v*. HC sample						Age-matched recAN *v*. HC sample					
	acAN (acAN-TP1)		HC		Test statistics		recAN		HC		Test statistics	
	*N*	M ± s.d.	*N*	M ± s.d.	*T*	*p*	*N*	M ± s.d.	*N*	M ± s.d.	*T*	*p*
Demographics												
Age (years)	82	16.83 ± 3.22	82	17.01 ± 3.22	0.35	0.729	20	22.66 ± 3.44	20	22.16 ± 3.18	−0.48	0.633
IQ	75	111.37 ± 12.50	82	111.23 ± 9.05	−0.08	0.937	20	111.85 ± 10.51	20	112.40 ± 7.42	0.19	0.849
SES^a^	71	3.28 ± 0.91	58	3.50 ± 0.94	1.38	0.171	19	3.13 ± 0.93	15	2.37 ± 0.52	−3.05	0.005**
BMI (kg/m^2^)	82	15.03 ± 1.23	82	20.72 ± 2.02	21.80	<0.001**	20	21.58 ± 1.52	20	21.17 ± 1.77	−0.79	0.432
BMI-SDS	82	−3.05 ± 1.19	82	−0.07 ± 0.66	19.75	<0.001**	20	−0.23 ± 0.54	20	−0.37 ± 0.60	−0.76	0.454
Minimal lifetime BMI (kg/m^2^)	81	14.61 ± 1.32	76	19.63 ± 1.82	19.69	<0.001**	19	14.59 ± 1.63	20	20.17 ± 1.75	10.31	<0.001**
Clinical measures												
EDI-2 core symptoms	81	25.52 ± 7.13	80	15.60 ± 4.78	−10.38	<0.001**	20	16.717 ± 4.732	19	14.211 ± 2.376	−2.105	0.044*
BDI-II total	82	24.15 ± 10.82	81	5.41 ± 6.02	−13.68	<0.001**	19	8.053 ± 7.989	20	3.1 ± 4.412	−2.379	0.024*
SCL-90-R GSI	82	1.07 ± 0.63	80	0.36 ± 0.40	−8.65	<0.001**	20	0.357 ± 0.285	19	0.242 ± 0.343	−1.136	0.263
Molecular factors												
IL-6 (μg/l)	82	1.06 ± 1.64	82	1.24 ± 1.63	NA	NA	20	0.96 ± 1.33	20	1.26 ± 1.75	NA	NA
Log(10)-IL-6	82	−0.23 ± 0.43	82	−0.12 ± 0.41	1.62	0.107	20	−0.17 ± 0.31	20	−0.11 ± 0.37	0.603	0.550
TNF-*α* (μg/l)	82	0.69 ± 0.30	82	0.74 ± 0.27	NA	NA	20	0.62 ± 0.23	20	0.65 ± 0.15	NA	NA
Log(10)-TNF-*α*	82	−0.19 ± 0.14	82	−0.15 ± 0.13	1.78	0.076	20	−0.23 ± 0.15	20	−0.20 ± 0.10	0.81	0.423
BDNF (μg/l)	66	20.65 ± 6.15	68	22.06 ± 6.54	NA	NA	7	23.70 ± 6.08	16	22.72 ± 5.89	NA	NA
Log(10)-BDNF	66	−0.02 ± 0.13	68	0.02 ± 0.10	1.49	0.138	7	0.02 ± 0.11	16	0.02 ± 0.09	−0.12	0.905
Neuromorphology												
eTIV (mm^3^)	82	1 490 324 ± 124 406	82	1 520 318 ± 113 172	1.61	0.108	20	1 514 783 ± 109 159	20	1 505 061 ± 136 471	−0.25	0.805
Subcortical GM volume (mm^3^)	82	57 713 ± 4826	82	61 678 ± 4327	5.54	<0.001**	20	62 559 ± 5281	20	63 890 ± 5163	0.81	0.425

AN, anorexia nervosa; BDI-II, Beck Depression Inventory-2; BDNF, brain-derived neurotrophic factor; BMI, body mass index; BMI-SDS, BMI standard deviation score; EDI-2, Eating Disorders Inventory-2; eTIV, estimated total intracranial volume; GM, grey matter; IL-6, interleukin-6; IQ, intelligence quotient; M, mean; SCL-90-R GSI, Symptom Checklist-90-Revised Global Severity Index; s.d., standard deviation; SES, socioeconomic status; TNF-*α*, tumor necrosis factor-alpha; TP, time-point.

In the acAN-TP1 group, comorbid psychiatric diagnoses included depressive disorders (*n* = 7), anxiety disorders (*n* = 3), obsessive-compulsive disorder (*n* = 1), Tourette syndrome (*n* = 2), adjustment disorders (*n* = 1), personality disorders (*n* = 1), developmental disorders (*n* = 1), and other psychiatric disorders (*n* = 3). No participants in the recAN group had a comorbid psychiatric disorder.

a SES was determined according to the parental (household) educational level/occupation group (range: 0 [lowest], leaving school without graduation – 5 [highest], graduating from university) (Patrick et al., 2004), given most study participants were adolescent, current students at school, university, or professional training institutions and still lived with their parents or guardians.

**Significant at the *p* < 0.01 threshold. *Significant at the *p* < 0.05 threshold.

#### Correlations between cytokines and BDNF

No associations emerged between BDNF and IL-6 or TNF-*α*, both cross-sectionally (whole sample, and AN/HC groups separately) and longitudinally. Online Supplementary Table S7 displays the full results of the Spearman's correlations.

#### Cross-sectional differences in hippocampal (sub-)regions

The results of the cross-sectional comparisons between acAN-TP1 and HC, and recAN and HC, are summarized in online Supplementary Tables S1 and S2, respectively.

Global hippocampal volumes were bilaterally smaller in acAN-TP1 compared with HC, as well as the hippocampal head and tail bilaterally, the right hippocampal body, and 19 hippocampal subfields (23 regions overall after FDR correction). Of these subfields, six were smaller in both hemispheres in acAN-TP1, including the CA1 head, the CA4 head, the GC ML DG head, the HATA, and the molecular layer HP body and head. In the right hemisphere, the CA1 body, CA3 head, CA4 body, GC ML DG body, presubiculum body and head, and subiculum head were smaller in AN. The only region larger in AN was the hippocampal fissure (a cerebral spinal fluid space) in the left hemisphere.

Three hippocampal subfields were larger in the recAN group compared with HC (none after FDR correction), including the fimbria in the left hemisphere, and the parasubiculum and presubiculum body in the right hemisphere.

#### Association between TNF-*α*, IL-6, and BDNF and cross-sectional differences in hippocampal (sub-)regions

The 27 regions of the hippocampus different between acAN-TP1 and HC were selected to investigate the effect of differences in TNF-*α*, IL-6, and BDNF on between-group differences (Table 2). There was no evidence for an interaction between concentrations of IL-6, TNF-*α*, or BDNF and group in any region of the hippocampus. For the recAN and HC comparisons, TNF-*α*, IL-6, and BDNF also had no effect on between-group differences in the three hippocampal subfields entered into analyses (see Table 3). The main effects of molecule for both comparisons can be viewed in online Supplementary Tables S3 and S4.

**Table 2.** Interaction effect between group and concentrations of TNF-*α*, IL-6 or BDNF on the volume of hippocampal regions (*n* = 27) volumetrically different between acAN-TP1 and controls, after controlling for age, age^2^ and eTIV

Region	TNF-*α*						IL-6						BDNF					
	*B*	s.e.	*t*	*p*	*q*	*d*	*B*	s.e.	*t*	*p*	*q*	*d*	*B*	s.e.	*t*	*p*	*q*	*d*
Left hemisphere																		
Whole hippocampus	−179.72	140.48	−1.28	0.203	0.355	0.20	42.55	46.64	0.91	0.363	0.494	0.15	−241.11	172.64	−1.40	0.165	0.452	0.25
Hippocampal head	−81.22	82.07	−0.99	0.324	0.464	0.16	26.14	26.97	0.97	0.334	0.464	0.15	−111.72	102.87	−1.09	0.279	0.570	0.19
Hippocampal tail	−43.70	38.86	−1.13	0.262	0.413	0.18	14.99	12.90	1.16	0.247	0.354	0.19	−47.18	45.84	−1.03	0.305	0.570	0.18
CA1 head	−27.05	27.65	−0.98	0.329	0.468	0.16	14.54	9.06	1.61	0.110	0.207	0.26	−27.40	34.32	−0.80	0.426	0.719	0.14
CA4 head	−1.20	7.78	−0.16	0.877	0.887	0.02	0.28	2.53	0.11	0.911	0.931	0.02	−12.72	9.60	−1.33	0.187	0.468	0.24
GC ML DG head	−2.85	9.30	−0.31	0.760	0.830	0.05	1.09	3.03	0.36	0.720	0.806	0.06	−11.13	11.45	−0.97	0.333	0.595	0.17
Fissure	8.96	13.53	0.66	0.509	0.645	0.11	−7.18	4.42	−1.62	0.107	0.203	0.26	−19.75	15.82	−1.25	0.214	0.515	0.22
HATA	−7.06	4.74	−1.49	0.139	0.280	0.24	1.12	1.58	0.71	0.481	0.618	0.11	−1.05	5.60	−0.19	0.852	0.895	0.03
Molecular layer HP body	−7.87	11.86	−0.66	0.508	0.645	0.11	0.76	3.93	0.19	0.846	0.888	0.03	−24.20	13.99	−1.73	0.086	0.271	0.31
Molecular layer HP head	−19.28	16.24	−1.19	0.237	0.397	0.19	5.09	5.38	0.95	0.345	0.477	0.15	−19.96	20.49	−0.97	0.332	0.595	0.17
Right hemisphere																		
Whole hippocampus	−153.66	146.16	−1.05	0.295	0.442	0.17	74.90	47.94	1.56	0.120	0.218	0.25	−186.08	177.28	−1.05	0.296	0.570	0.19
Hippocampal body	−35.16	56.39	−0.62	0.534	0.664	0.10	16.32	18.55	0.88	0.381	0.507	0.14	−67.08	65.59	−1.02	0.308	0.570	0.18
Hippocampal head	−92.17	81.09	−1.14	0.257	0.409	0.18	36.51	26.64	1.37	0.172	0.273	0.22	−66.33	101.53	−0.65	0.515	0.791	0.12
Hippocampal tail	−26.33	40.03	−0.66	0.512	0.645	0.10	22.08	13.14	1.68	0.095	0.185	0.27	−52.68	47.60	−1.11	0.271	0.570	0.20
CA1 body	5.79	11.75	0.49	0.623	0.736	0.08	4.50	3.80	1.18	0.238	0.343	0.19	−13.32	14.22	−0.94	0.351	0.619	0.17
CA1 head	−23.79	30.10	−0.79	0.431	0.578	0.13	15.72	9.83	1.60	0.112	0.207	0.26	−31.28	37.40	−0.84	0.404	0.695	0.15
CA3 head	−7.72	9.77	−0.79	0.431	0.578	0.13	−1.78	3.19	−0.56	0.577	0.700	0.09	−4.17	11.99	−0.35	0.728	0.867	0.06
CA4 body	−3.18	8.00	−0.40	0.692	0.783	0.06	0.26	2.64	0.10	0.921	0.936	0.02	−4.13	9.34	−0.44	0.659	0.853	0.08
CA4 head	−4.22	7.74	−0.55	0.587	0.711	0.09	0.23	2.53	0.09	0.929	0.936	0.01	−3.80	9.66	−0.39	0.695	0.867	0.07
GC ML DG body	−3.27	7.61	−0.43	0.668	0.766	0.07	1.00	2.50	0.40	0.691	0.798	0.06	−4.31	8.80	−0.49	0.625	0.830	0.09
GC ML DG head	−5.82	9.85	−0.59	0.556	0.686	0.09	0.45	3.22	0.14	0.889	0.918	0.02	−4.14	12.25	−0.34	0.736	0.867	0.06
HATA	−5.49	5.25	−1.05	0.297	0.443	0.17	1.24	1.74	0.71	0.478	0.618	0.11	−3.03	6.49	−0.47	0.641	0.842	0.08
Molecular layer HP body	−3.90	13.22	−0.30	0.768	0.833	0.05	5.61	4.30	1.31	0.194	0.298	0.21	−17.79	15.44	−1.15	0.251	0.546	0.20
Molecular layer HP head	−17.99	16.25	−1.11	0.270	0.418	0.18	7.65	5.36	1.43	0.156	0.265	0.23	−14.01	20.40	−0.69	0.494	0.776	0.12
Presubiculum body	−10.45	10.50	−1.00	0.321	0.464	0.16	2.82	3.47	0.81	0.418	0.549	0.13	4.19	12.73	0.33	0.743	0.867	0.06
Presubiculum head	−9.73	7.44	−1.31	0.193	0.351	0.21	3.32	2.47	1.35	0.180	0.282	0.21	−2.39	8.94	−0.27	0.790	0.889	0.05
Subiculum head	−11.12	13.23	−0.84	0.402	0.558	0.13	6.34	4.37	1.45	0.149	0.263	0.23	−8.10	16.04	−0.51	0.614	0.827	0.09

*B*, unstandardized *β*; BDNF, brain-derived neurotrophic factor; CA, cornu ammonis; *d*, Cohen's *d*; GC ML DG, granule cell and molecular layer of the dentate gyrus; HATA, hippocampus-amygdala-transition-area; HP, hippocampus; IL-6, interleukin-6; *q*, False Discovery Rate-adjusted *p*-value; TNF-*α*, tumor necrosis factor-alpha.

Reported statistics refer to the group × molecule interaction term.

*Significant at the *p* < 0.05 threshold.

**Table 3.** Interaction effect between group and concentrations of TNF-*α*, IL-6, or BDNF on the volume of hippocampal regions (*n* = 3) volumetrically different between recAN and controls, after controlling for age, age^2^, and eTIV

Region	TNF-*α*						IL-6						BDNF					
	*B*	s.e.	*t*	*p*	*q*	*d*	*B*	s.e.	*t*	*p*	*q*	*d*	*B*	s.e.	*t*	*p*	*q*	*d*
Left hemisphere																		
Fimbria	4.026	20.72	0.19	0.847	0.847	0.07	3.60	7.21	0.50	0.621	0.811	0.17	47.02	37.55	1.25	0.229	0.343	0.63
Right hemisphere																		
Parasubiculum	−4.60	14.84	−0.31	0.758	0.847	0.11	−1.25	5.18	−0.24	0.811	0.811	0.08	3.65	25.28	0.15	0.887	0.887	0.07
Presubiculum body	−8.50	22.42	−0.38	0.707	0.847	0.13	−2.91	7.74	−0.38	0.710	0.811	0.13	57.52	32.90	1.75	0.100	0.299	0.87

*B*, Unstandardized *β*; BDNF, brain-derived neurotrophic factor; *d*, Cohen's *d*; FDR, false discovery rate; IL-6, interleukin-6; *q*, False Discovery Rate-adjusted *p*-value; TNF-*α*, tumor necrosis factor-alpha.

Reported statistics refer to the group × molecule interaction term.

### Longitudinal results

#### Demographic and clinical characteristics, cytokines, global GM

The demographic and clinical characteristic comparisons in acAN longitudinally from TP1 to TP2 are summarized in Table 4. The mean ± s.d. interval between TP1 and TP2 was 88 ± 27 days. Over this timeframe, age, BMI and BMI-SDS, and subcortical GM volumes significantly increased, whereas depression (BDI-II), overall psychological distress (SCL-90-R GSI) but not ED symptoms (EDI-2) decreased. No changes in IL-6 and TNF-*α* were observed but BDNF increased, which remained the same when removing patients on antidepressants from the sample (online SM 2.2.1). When removing patients with the AN-BP subtype, the increase in BDNF was reduced to a statistical trend.

**Table 4.** Demographic and clinical measures from longitudinal sample

Variable	Longitudinal samples					Test statistics		
	acAN-TP1		acAN-TP2		Change			
	*N*	M ± s.d.	*N*	M ± s.d.	88 ± 27 days	*F*	df	*p*
Demographics								
Age (years)	58	16.44 ± 2.27	58	16.67 ± 2.25	0.23 ± 0.07	669.21	1;57	<0.001**
IQ	55	112.51 ± 13.02	NA	NA	NA	NA	NA	NA
SES^a^	49	3.27 ± 0.88	NA	NA	NA	NA	NA	NA
BMI (kg/m^2^)	58	15.14 ± 1.13	58	19.18 ± 1.02	4.04 ± 1.04	805.46	1;48	<0.001**
BMI-SDS	58	−2.92 ± 0.95	58	−0.65 ± 0.54	2.27 ± 0.74	602.33	1;71	<0.001**
Minimal lifetime BMI (kg/m^2^)	57	14.87 ± 1.14	NA	NA	NA	NA	NA	NA
Clinical measures								
EDI-2 core symptoms	57	24.89 ± 7.35	58	23.71 ± 8.14	−1.16 ± 6.29	2.37	1;80	0.127
BDI-II total	58	22.76 ± 10.86	58	14.75 ± 10.89	−8.01 ± 10.37	45.06	1;91	<0.001**
SCL-90-R GSI	58	0.99 ± 0.61	57	0.68 ± 0.56	−0.31 ± 0.52	25.21	1;79	<0.001**
Molecular factors								
IL-6 (μg/l)	58	1.12 ± 1.77	58	1.16 ± 1.55	0.04 ± 1.15	NA	NA	NA
Log(10)-IL-6	58	−0.24 ± 0.47	58	−0.18 ± 0.44	0.06 ± 0.37	1.58	1;64	0.213
TNF-*α* (μg/l)	58	0.67 ± 0.20	58	0.69 ± 0.26	0.02 ± 0.20	NA	NA	NA
Log(10)-TNF-*α*	58	−0.19 ± 0.12	58	−0.18 ± 0.14	0.01 ± 0.11	0.12	1;60	0.731
BDNF (μg/l)	45	20.07 ± 6.31	44	21.89 ± 6.44	1.75 ± 4.33	NA	NA	NA
Log(10)-BDNF	45	−0.03 ± 0.13	44	0.01 ± 0.12	0.03 ± 0.10	4.88	1;51	0.032*
Neuromorphology								
eTIV (mm^3^)	58	1 437 364 ± 119 391	NA	NA	NA	NA	NA	NA
Subcortical GM volume (mm^3^)	58	61 733 ± 4898	58	63 639 ± 4829	1906 ± 1282	131.76	1;63	<0.001**

AN, anorexia nervosa; BDI-II, Beck Depression Inventory-2; BDNF, brain-derived neurotrophic factor; BMI, body mass index; BMI-SDS, BMI standard deviation score; EDI-2, Eating Disorders Inventory-2; eTIV, estimated total intracranial volume; GM, grey matter; IL-6, interleukin-6; IQ, intelligence quotient; M, mean; SCL-90-R GSI, Symptom Checklist-90-Revised Global Severity Index; s.d., standard deviation; SES, socioeconomic status; TNF-*α*, tumor necrosis factor-alpha; TP, time-point.

In the acAN-TP1 group, comorbid psychiatric diagnoses included depressive disorders (*n* = 3), anxiety disorders (*n* = 1), Tourette syndrome (*n* = 2), adjustment disorder (*n* = 1), personality disorders (*n* = 2), and other psychiatric disorders (*n* = 2).

a SES was determined according to the parental (household) educational level/occupation group (range: 0 [lowest], leaving school without graduation – 5 [highest], graduating from university) (Patrick et al., 2004), given most study participants were adolescent, current students at school, university, or professional training institutions and still lived with their parents or guardians.

**Significant at the *p* < 0.01 threshold. *Significant at the *p* < 0.05 threshold.

#### Differences in hippocampal (sub-)regions between AN-TP1 and AN-TP2

Both the right and left global hippocampi as well as the bilateral hippocampal tail, the left hippocampal head (before FDR), and seven hippocampal subfields (after FDR five subfields, see online Supplementary Table S5 and SM 2.2.2) increased in volume as a linear function of increases in BMI-SDS. Regions that were larger at TP2 in the left hemisphere include the GC ML DG head, molecular layer HP body, molecular layer HP head, and presubiculum body. In the right hemisphere, the hippocampal–amygdala transition area, molecular layer HP body, and presubiculum body were larger at TP2. In both hemispheres, the CA3 body and fissure were smaller at TP2.

#### Association between log10 ΔTNF-*α*, ΔIL-6, and ΔBDNF and longitudinal hippocampal changes over and above ΔBMI-SDS

The 16 regions of the hippocampus different in acAN between TP1 and TP2 were investigated for the effect of ΔTNF-*α*, ΔIL-6, and ΔBDNF on longitudinal differences (Table 5). ΔTNF-*α* and ΔIL-6 had no effect on longitudinal differences in any region above and beyond the BMI-SDS effects. However, ΔBDNF had a positive effect above and beyond the increasing BMI on changes in both the left and right whole hippocampus. After removing patients on anti-depressant medication (*n* = 4) or those with the AN-BP subtype (*n* = 11), results remained largely unchanged (online Supplementary Table S6 and SM 2.2.3.).

**Table 5.** The contribution of ΔTNF-*α*, ΔIL-6, and ΔBDNF to longitudinal differences in (*n* = 16) hippocampal (sub-)regions between TP1 and TP2 in patients with AN after controlling for age, age^2^, eTIV, and ΔBMI-SDS

Region	TNF-*α*						IL-6						BDNF					
	*B*	s.e.	*t*	*p*	*q*	*d*	*B*	s.e.	*t*	*p*	*q*	*d*	*B*	s.e.	*t*	*p*	*q*	*d*
Left hemisphere																		
Whole hippocampus	28.34	95.53	0.30	0.768	0.768	0.04	−5.56	28.27	−0.20	0.845	0.845	0.03	330.43	119.29	2.77	0.008**	0.016*	0.38
Whole hippocampal head	16.28	45.99	0.35	0.725	0.901	0.05	−2.71	13.60	−0.20	0.843	0.976	0.03	113.90	51.64	2.21	0.033*	0.230	0.30
Hippocampal tail	62.17	36.62	1.70	0.095	0.505	0.22	−6.09	11.18	−0.54	0.588	0.976	0.07	144.93	47.16	3.07	0.004**	0.050	0.42
CA3 body	−5.78	6.87	−0.84	0.403	0.678	0.11	0.82	2.04	0.40	0.689	0.976	0.05	0.06	9.83	0.01	0.996	0.996	<0.01
GC ML DG head	−1.89	6.48	−0.29	0.772	0.901	0.04	1.22	1.91	0.64	0.525	0.976	0.08	13.43	8.05	1.67	0.102	0.267	0.23
Hippocampal fissure	−21.88	21.57	−1.01	0.314	0.628	0.13	0.94	6.48	0.15	0.885	0.976	0.02	8.93	24.73	0.36	0.720	0.894	0.05
Molecular layer HP body	−14.36	10.60	−1.35	0.180	0.505	0.18	0.99	3.18	0.31	0.756	0.976	0.04	15.09	14.87	1.01	0.315	0.441	0.14
Molecular layer HP head	1.54	10.62	0.15	0.885	0.949	0.02	0.45	3.14	0.14	0.887	0.976	0.02	19.62	12.18	1.61	0.115	0.267	0.22
Presubiculum body	−8.72	11.12	−0.78	0.436	0.678	0.10	1.46	3.29	0.44	0.660	0.976	0.06	17.18	15.14	1.13	0.263	0.408	0.15
Right hemisphere																		
Whole hippocampus	−89.14	79.09	−1.13	0.264	0.529	0.15	−16.56	23.57	−0.70	0.485	0.845	0.09	182.70	85.65	2.13	0.039*	0.039*	0.29
Hippocampal tail	−40.68	33.03	−1.23	0.223	0.520	0.16	−1.87	9.89	−0.19	0.851	0.976	0.02	71.68	38.51	1.86	0.069	0.267	0.25
CA3 body	−9.85	6.10	−1.61	0.112	0.505	0.21	−1.57	1.83	−0.86	0.393	0.976	0.11	−1.21	8.06	−0.15	0.881	0.949	0.02
HATA	0.26	4.08	0.06	0.949	0.949	0.01	−1.58	1.20	−1.32	0.193	0.976	0.17	−6.06	4.48	−1.35	0.183	0.320	0.18
Hippocampal fissure	−35.00	22.01	−1.59	0.116	0.505	0.21	0.20	6.63	0.03	0.976	0.976	<0.01	7.70	25.82	0.30	0.767	0.894	0.04
Molecular layer HP body	−5.82	9.54	−0.61	0.545	0.762	0.08	0.19	2.82	0.07	0.947	0.976	0.01	20.55	12.72	1.62	0.113	0.267	0.22
Presubiculum body	13.75	10.11	1.36	0.179	0.505	0.18	3.72	3.01	1.24	0.220	0.976	0.16	19.21	13.72	1.40	0.168	0.320	0.19

*B*, unstandardized *β*; BDNF, brain-derived neurotrophic factor; CA, cornu ammonis; *d*, Cohen's *d*; GC ML DG, granule cell and molecular layer of the dentate gyrus; HATA, hippocampus-amygdala-transition-area; HP, hippocampus; IL-6, interleukin-6; *q*, False Discovery Rate-adjusted *p*-value; TNF-*α*, tumor necrosis factor-alpha.

**Significant at the *p* < 0.01 threshold. *Significant at the *p* < 0.05 threshold.

## Discussion

This study aimed to investigate associations between pro-inflammatory cytokines (IL-6 and TNF-*α*), a neurotrophin (BDNF), and reductions followed by subsequent increases in whole and sub-regional hippocampal volumes in adolescent/young adult patients with AN. Aligning with prior research (Collantoni et al., 2021; Keeler et al., 2020; Myrvang et al., 2018), this study found widespread reductions in hippocampal volumes in patients with acAN-TP1 compared with HC. There were bilateral reductions in several subfields of the hippocampal head, but also reductions in additional subfields of only the right hemispheric hippocampal head (e.g. CA3, presubiculum) and body (e.g. CA1, presubiculum, granule cell, and molecular layer of the dentate gyrus). Thus, to some degree, more regions of the hippocampus in the right hemisphere of the brain in people with AN were volumetrically reduced than in the left hemisphere. After partial weight restoration (i.e. from TP1 to TP2), generalized bilateral increases in hippocampal volumes were found, although increases were not found in many hippocampal subfields that were reduced cross-sectionally. There were no robust differences in hippocampal subfield volumes between recAN and HC. Contrary to prediction, there were no group differences between acAN-TP1 and HC in peripheral concentrations of cytokines and BDNF. Cross-sectionally, there were no associations between concentrations of either cytokine or BDNF and between-group differences in hippocampal subfield volumes. Longitudinally, IL-6 and TNF-*α* during weight restoration treatment did not change and were not associated with increases in hippocampal (sub-)field volumes. However, increases in serum BDNF in patients with AN were positively associated with increasing bilateral global hippocampal volumes longitudinally, beyond the effect of increases in BMI in patients with AN.

In the consideration of our findings, it is important to note that concentrations of IL-6, TNF-*α*, and BDNF were not found to be different between acAN-TP1 and HC, partially contrasting with the previous literature (Dalton et al., 2018; Keeler et al., 2022) but aligning with more recent research findings that account for potentially confounding variables (e.g. smoking status, psychotropic medication; Keeler et al., 2021a; Specht et al., 2022). Moreover, concentrations of IL-6 did not decrease with weight restoration, contrasting with a prior study (Dalton et al., 2020). Therefore, the lack of evidence for heightened inflammation in our sample may drive these null findings.

Our findings indicate that increases in BDNF, when controlling for increases in BMI, contribute to global hippocampal recovery in AN, although BDNF does not explain the reduced hippocampal volume found in the acute underweight state. These findings align with a meta-analysis, which found increases in BDNF in people with AN following treatment (Keeler et al., 2022), although BDNF levels have been found to be unaffected in adolescents with AN (Steinhäuser et al., 2021). It is generally accepted that mature BDNF promotes neuroplasticity in the hippocampus and contributes to hippocampal volume (Erickson, Miller, & Roecklein, 2012; Lu, Pang, & Woo, 2005), which supports learning and therefore perhaps bolsters a successful outcome following psychological intervention. It is unclear what causes increases in BDNF over time in AN, although it has been suggested that the increased availability of energy during nutritional reinstatement (and associated changes to the gut microbiome [Herpertz-Dahlmann, Seitz, & Baines, 2017]) may be instrumental rather than simple weight gain (Keeler et al., 2022). Alternatively, it is possible that during recovery from AN, psychological stress associated with the illness is ameliorated, which may increase BDNF levels mediated by a reduction in corticosterone (Duman & Monteggia, 2006). However, in our study, ED psychopathology did not significantly reduce following treatment. Nevertheless, novel therapeutics (e.g. ketamine [Keeler, Kan, Treasure, & Himmerich, 2023; Keeler, Treasure, Juruena, Kan, & Himmerich, 2021b]) may target BDNF levels in people with AN in order to bolster neuroplasticity and learning, although it should be noted that BDNF has been documented to have suppressive effects on appetite (Rios, 2013; Stanek et al., 2008), which is undesirable in this population (Trinh, Keller, Herpertz-Dahlmann, & Seitz, 2023).

### Strengths and limitations

This study utilized a relatively homogenous sample, in terms of sex, IQ, and eTIV across acAN, recAN, and HC, using a standardized protocol and the same scanner for sMRI acquisition. Age was controlled for in all statistical models and through age-matching of the cross-sectional samples. However, although our sample is representative of the local female population, the diversity of the sample was limited in regard to gender, ethnicity, and race, as well as age categories. Thus, the findings cannot be generalized fully to adults, non-females, or non-white Europeans. In particular, the developmental trajectories of BDNF levels (Kowiański et al., 2018), as well as of hippocampal structure, function, and recruitment (Tottenham & Sheridan, 2010) complicate comparisons between adolescent and adult populations and generalizability. Additionally, the recAN group was small, meaning that this cross-sectional analysis may be underpowered.

Another strength of this study is the segmentation of a large number of hippocampal subfields in each hemisphere individually, thereby allowing us to explore potentially hemisphere-specific effects. Notably, some of the subfields of the hippocampus are included in the ‘whole hippocampal head’ or ‘whole hippocampal body’, as mentioned in the methods, meaning that our correction for multiple testing may have been too conservative. Moreover, in the segmentation of the hippocampus using Freesurfer, there is evidence of poor spatial reliability in some subfields of the hippocampus, such as the CA3 head and fissure, whilst numerical reliability has been found to be reasonable (Kahhale, Buser, Madan, & Hanson, 2021). Cautious interpretation of Freesurfer outputs has been recommended (Kahhale et al., 2021). However, the numerical and spatial reliabilities of larger regions central to our main findings have been found to be reasonable (Kahhale et al., 2021; Quattrini et al., 2020).

It should also be noted that the analytical approaches used in the present study facilitate associations and therefore do not enable the establishment of cause and effect. Whilst we tested the influence of weight gain in the longitudinal analyses, it is possible that a third unmeasured variable could be contributing to the observed findings.

Finally, it is unclear whether peripheral levels of cytokines align to central (i.e. brain) levels, and more widely, whether inflammatory processes in the central and peripheral nervous systems occur synchronously. Thus, it is conceivable that there could be differences between peripheral (i.e. serum or plasma) concentrations of cytokines and BDNF, and central (i.e. cerebrospinal fluid) concentrations. However, rodent studies indicate that serum levels of BDNF align with cortical and hippocampal levels (Karege, Schwald, & Cisse, 2002; Sartorius et al., 2009).

## Conclusions

The findings of this study indicate a role for the neurotrophin BDNF, but not for the pro-inflammatory cytokines TNF-*α* and IL-6, in promoting increases in global hippocampal volumes in adolescent/young adult AN patients after partial weight restoration, over and above increases in weight. Our findings suggest that increases in BDNF may be a biological correlate of increases in hippocampal volumes during weight restoration, although these effects are not subfield-specific. If further research replicates our findings, increases in BDNF may emerge as a target for the development of novel pharmacological treatments, which may improve neuroplasticity and learning, and support the potential for psychotherapeutic success in people with AN.

## Supporting information

Keeler et al. supplementary material Keeler et al. supplementary material

## Supplementary material

The supplementary material for this article can be found at https://doi.org/10.1017/S0033291724000394
