# Supplementary material for: Longitudinal changes in brain-derived neurotrophic factor (BDNF) but not cytokines contribute to hippocampal recovery in anorexia nervosa above increases in body mass index
Source: Psychol Med. 2024 Mar 7;54(9):2242–53. doi: 10.1017/S0033291724000394 (PMC11413355; doi:10.1017/S0033291724000394)
Supplement: Keeler et al. supplementary material [file S0033291724000394sup001.docx]

Appendix: Supplementary Materials for “Longitudinal changes in brain-derived neurotrophic factor (BDNF) but not cytokines contribute to hippocampal recovery in anorexia nervosa above increases in body mass index”

**Authors**

Johanna Louise Keeler ^a^ (johanna.keeler@kcl.ac.uk) (J.L.K.)

Klaas Bahnsen.^b^ (Klaas.Bahnsen@uniklinikum-dresden.de) (K.B.)

Marie-Louis Wronski ^b,c^ (Marie-Louis.Wronski@uniklinikum-dresden.de) (M.W.)

Fabio Bernardoni ^b^ (Fabio.Bernardoni@uniklinikum-dresden.de) (F.B.)

Friederike Tam ^b,d^ (Friederike.Tam@uniklinikum-dresden.de) (F.T.)

Dominic Arold ^b^ (Dominic.Arold@uniklinikum-dresden.de) (D.A.)

Joseph A. King ^b^ (Joseph.King@uniklinikum-dresden.de) (J.A.K.)

Theresa Kolb ^b^ (Theresa.Kolb@uniklinikum-dresden.de) (T.K.)

David Poitz ^e^ (David.Poitz@uniklinikum-dresden.de) (D.P.)

Veit Roessner ^f^ (Veit.Roessner@uniklinikum-dresden.de) (V.R.)

Janet Treasure ^a^ (janet.treasure@kcl.ac.uk) (J.T.)

Hubertus Himmerich ^a^ (hubertus.himmerich@kcl.ac.uk) (H.H.)

Stefan Ehrlich, MD, PhD ^b,d^ (transden.lab@uniklinikum-dresden.de) (S.E.)

^a^ Department of Psychological Medicine, Institute of Psychiatry, Psychology & Neuroscience, King’s College London, London, UK.

^b^ Translational Developmental Neuroscience Section, Division of Psychological and Social Medicine and Developmental Neurosciences, Faculty of Medicine, TU Dresden, Dresden, Germany.

^c^ Neuroendocrine Unit, Department of Medicine, Massachusetts General Hospital and Harvard Medical School, Boston, MA, USA.

^d^ Eating Disorder Treatment and Research Center, Department of Child and Adolescent Psychiatry, Faculty of Medicine, TU Dresden, Dresden, Germany.

^e^ Institute for Clinical Chemistry and Laboratory Medicine, TU Dresden, Dresden, Germany.

^f^ Clinic and Polyclinic for Pediatric and Adolescent Medicine, Faculty of Medicine, TU Dresden, Dresden, Germany.

Correspondence to Stefan Ehrlich, Division of Psychological and Social Medicine and Developmental Neurosciences, Faculty of Medicine, TU Dresden, Fetscherstraße 74, 01307 Dresden, Germany, Tel: +49 351 458-5214, Fax: +49 351 458-7206, E-mail: transden.lab@uniklinikum-dresden.de.

**SM 1 Supplemental Methodology**

**SM 1.1 Clinical measures**

The expert form of the Structured Interview for Anorexia and Bulimia Nervosa (SIAB-EX) (M. M. Fichter & Quadflieg, 1999), a well-validated 87-item semi-standardized interview with good inter-rater reliability (M. Fichter & Quadflieg, 2001), was used to evaluate the presence and severity of lifetime (from onset of puberty) or current (within 3 months prior to the study) eating-related psychopathology (including assessment of AN subtype and physical activity used for descriptive statistics). SIAB-EX interviews were conducted by clinically experienced and trained research assistants under the supervision of a child and adolescent psychiatrist. Co-existing psychiatric diagnoses other than eating disorders were derived according to standard practice from medical records and confirmed by a board licensed child and adolescent psychiatrist with over 10 years of clinical experience.

**SM 1.2. Additional Measures**

Other pertinent information was collected from all participants using our own semi-structured research interview which includes several speciﬁc questions to assess menstruation history, weight history, general medical and pharmaceutical history, family psychiatric history, smoking status, and socioeconomic factors (e.g., educational level, occupation, family status, current living situation).

**SM 1.3. Structural MRI Image Data Processing**

High-resolution three-dimensional T1-weighted structural scans were acquired on a Siemens Magnetom Trio 3T Scanner with a magnetization-prepared rapid gradient-echo (MP-RAGE) sequence using the same parameters as in our previous studies (Bahnsen et al., 2022; Bernardoni et al., 2016): 176 sagittal slices (1mm thickness, no gap, TR=1900ms; TE=2.26ms; flip angle=9º; voxel size=1.0x1.0x1.0mm; FoV=256x224mm^2^; bandwidth of 200 Hz/pixel). Reconstruction of the cerebral cortex was accomplished automatically with FreeSurfer 7.1.1. (Desikan et al., 2006; Bruce Fischl et al., 2002). Next, preprocessed images were analyzed with FreeSurfer’s automated processing stream (Reuter, Schmansky, Rosas, & Fischl, 2012) and the cross-sectional and longitudinal processed scans were then analyzed with either a cross-sectional or longitudinal combined amygdala and hippocampus subsegmentation functionality (Saygin, Osher, Augustinack, Fischl, & Gabrieli, 2011) implemented in FreeSurfer 7.1.1. This functionality relies on a Bayesian probabilistic atlas to assign an anatomical label to each voxel (created from 7.0T post-mortem human MRI data and in-vivo training MRI data (Saygin et al., 2011)) and jointly sub-segments one or multiple time points per subject using subject-specific atlases and Bayesian inference to improve the robustness and sensitivity of the subsegmentation (Iglesias et al., 2016). Scans underwent a multi-stage standardized quality control procedure (see below section for further details). Participants with misapplied amygdala or hippocampal sub-segmentation were excluded (3.35% in total which is similar to previous hippocampal subfield studies (3.50%) (Reuter et al., 2012).

**SM 1.4. Quality Control (QC)**

We developed a visual and partly outlier-guided multi-stage QC procedure for combined amygdala and hippocampus subsegmentations in line with recently published recommendations for hippocampal subfield QC by the ENIGMA consortium (Sämann et al., 2022) and under expert consultation with two independent ENIGMA representatives (http://enigma.ini.usc.edu/). Our QC involved:

1. Overall scan quality: Images with significant downward deviation from the study group means of signal-to-noise ratio (SNR) and/or contrast-to-noise ratio (CNR) were *a-priori* excluded (scan quality measures SNR and CNR obtained from FreeSurfer’s recon-all output statistics).
2. General cortical and subcortical QC follows standardized procedures established within the ENIGMA consortium (http://enigma.ini.usc.edu/) and includes checks of the completeness of all FreeSurfer “recon-all” pipeline steps, visual inspection of HTML-snapshots of various surfaces and volumes, study group-wise statistical outlier detection, and dynamic visual investigation of images with artifacts and/or outliers using FreeView (B. Fischl, 2012). Participants with significant artifacts in general QC (e.g., skull inclusion or misapplied general subcortical segmentation) were excluded prior to amygdala and hippocampus subsegmentation QC.
3. Amygdala and hippocampus subsegmentation QC test phase in FreeView: Trained QC raters (N=2) viewed and independently rated the same 20 randomly selected images with amygdala and hippocampus subsegmentations from patient and control groups to determine inter-rater reliability, which was substantial (κ=0.76).
4. HTML-snapshot-based visual QC of all amygdala and hippocampus subsegmentations primarily checking whether 1) binary hippocampal mask was visible, 2) hippocampal fissure was located within the hippocampal mask, and 3) coloured amygdala and hippocampus segmentation overlays were complete. Observed artifacts were re-inspected via FreeView. Segmentation failures were excluded.
5. Dynamic FreeView-based visual QC: Images with artifacts in the preceding snapshot QC and/or statistical outliers were dynamically investigated in axial, coronal, and sagittal planes simultaneously for left and right brain hemispheres starting at the hippocampal fissure. FreeView QC focused on 1) colour allocation to/labelling of individual amygdala and hippocampus subregions, 2) large and unambiguous artifacts in the subsegmentation such as obliterations, blurred margins, or white matter inclusions, 3) bilateral subsegmentation symmetry, and 4) smaller artifacts such as spared regions (i.e., “holes”) in single subregions or merging neighbouring subregions. Study group-wise statistical outlier detection criteria for individual subregions were based on 1) subregion volume and 2) bilateral symmetry (lateralization index (Kong et al., 2022)). Quantitative outlier cut-offs for FreeView QC were defined as ≥1 “extreme” or ≥2 “mild” volume or symmetry outlier(s).
6. QC ratings: Subsegmentation quality was rated as either “1” for “inclusion” or “9” for “exclusion” of the entire amygdala and hippocampus subsegmentation of a study participant. Unclear cases were discussed in an expert QC meeting.

**SM 1.5. Adjustment of BDNF values for batch effects and storage time**

As recommended by previous publications (Steinhäuser et al., 2021; Steinhäuser, Wronski, Keeler, Ehrlich, & King, 2022), BDNF values were adjusted for batch effects and storage time in each sample separately; values of BDNF levels were predicted by batch and storage time using a multiple linear regression model: $BDNF=A+ B_{batch}+ C_{storage time}$. The residual BDNF values produced by this model, after adjusting for B (batch) and C (storage time), were used in analyses henceforth.

**SM 1.6. Statistical analysis plan**

- Multi-stage QC of hippocampus subsegmentations (and amygdala) 🡪 1 QC-approved scan/participant (1,031 MRI scans)
- Removing participants without cytokine and BDNF data (824 participants)
- Imputation of two non-detectable IL-6 values using $\frac{limit of detection}{\surd2}$.

**LONGITUDINAL**

- Adjustment of BDNF values for batch and storage time effects using multiple linear regression and residualization
- Log10 transformation of IL-6, TNF-α and BDNF
- Descriptive statistics (demographic and clinical variables) [MS Table 4]

**CROSS-SECTIONAL**

- Adjustment of BDNF values for batch and storage time effects using multiple linear regression and residualization
- Log10 transformation of IL-6, TNF-α and BDNF
- Age-matching of acAN-TP1 to HC (n=82 per sample) and of recAN to HC (n=20 per sample) for cross-sectional analyses using a minimized sum of absolute pairwise distances in the matched sample
- Descriptive statistics (demographic and clinical variables) [MS Table 1]

**3. MAIN ANALYSES**

- Demographic, cytokine, BDNF, total gray matter, eTIV and psychiatric symptom variables

**a) LME model: hippocampal (subfield) volume**

- Main LME model:

$$HV=A+\Delta_{recAN}+\Delta_{acAN}+ B_{acAN}\left( b_{t}-b_{TP2} \right)$$

$$+C_{acAN}\left( c_{t}-c_{TP2} \right)+D_{age}{+ E}_{age2}+F_{eTIV}$$

- Significant regions (prior to FDR correction, n=16) taken forward to next stage

**a) GLM model: hippocampal (subfield) volume**

- Main GLM model:

$$HV=A+B_{acAN}+B_{recAN}+ C_{age}{+ D}_{age2}+E_{eTIV}$$

- In acAN-TP1 vs. HCs (n=82 per group) 🡨 FDR-adjustment across all tested hippocampal (sub-)regions
- In recAN-TP1 vs. HCs (n=20 per group) 🡨 FDR-adjustment across all tested hippocampal (sub-)regions
- Regions significantly different (prior to FDR correction) between acAN and HC (n=27) or recAN and HC (n=3) taken forward to next stage

**b) LME model: adding cytokines/BDNF**

- LME including TNF-α, IL-6 or BDNF:

$$HV=A+\Delta_{recAN}+\Delta_{acAN}+ B_{acAN}\left( b_{t}-b_{TP2} \right)+D_{acAN}\left( d_{t}-d_{TP2} \right)+C age$$

- Contrasts used: acAN-TP2-acAN-TP1 🡨 FDR-adjustment across all contrasts and tested hippocampal (sub-)regions

**b) GLM model: adding cytokines/BDNF**

- GLM including TNF-α, IL-6 or BDNF:

$$HV=A+B_{acAN}+B_{recAN}+ C_{age}{+ D}_{age2}+ E_{molecule}+ F_{molecule*acAN}+ G_{molecule*recAN}$$

- FDR-adjustment across all tested hippocampal (sub-)regions

**4. SUPPLEMENTARY ANALYSES**

- Demographic, cytokine, BDNF, total gray matter, eTIV and psychiatric symptom variables

**LME model b) removing cases:**

- With AN-BP subtype (n=11)
- On antidepressant medication (n=4)

**ANOVAs**

- Demographic, cytokine, BDNF, total gray matter, eTIV and psychiatric symptom variables

**2. INITIAL ANALYSES**

- Demographic, cytokine, BDNF, total gray matter, eTIV and psychiatric symptom variables

**T-tests**

- Demographic, cytokine, BDNF, total gray matter, eTIV and psychiatric symptom variables

**Spearman’s correlations between**:

- $\Delta$BDNF and $\Delta$IL-6
- $\Delta$BDNF and $\Delta$TNF-α

**Spearman’s correlations in AcAN-TP1 and HC sample individually between**:

- BDNF and IL-6
- BDNF and TNF-α

**1. PRELIMINARIES**

**SM 2 Supplemental Results**

**SM 2.1. Cross-sectional results**

*SM 2.1.1. Demographic and clinical characteristics excluding smokers, patients with the AN-BP subtype and participants taking anti-depressant medication*

After removing participants who smoke between acAN-TP1 (n=1) and HC (n=5), there remained no between-group differences in BDNF (*t*(128)=1.06; *p*=0.289), IL-6 (*t*(156)=1.40; *p*=0.162) or TNF-α (*t*(156)=1.76; *p*=0.081). After removing participants who smoke between recAN (n=3) and HC (n=2), there were likewise no between-group differences in BDNF (*t*(18)=0.05; *p*=0.960), IL-6 (*t*(33)=0.56; *p*=0.579) or TNF-α (*t*(33)=0.63; *p*=0.534).

After removing acAN-TP1 participants with the AN-BP subtype (n=11), there remained no differences compared to HC in BDNF (*t*(125)=1.07; *p*=0.285), IL-6 (*t*(151)=1.54; *p*=0.126) or TNF-α (*t*(151)=1.43; *p*=0.153). Likewise, when removing recAN cases with a previous subtype of AN-BP (n=1), there were no differences in BDNF (*t*(20)=-0.13; *p*=0.900), IL-6 (*t*(37)=0.61; *p*=0.546) or TNF-α (*t*(37)=0.98; *p*=0.334) compared with HC.

When removing acAN-TP1 participants on anti-depressant medication (n=3), there were no differences compared to HC in concentrations of BDNF (*t*(129)=1.52; *p*=0.131) or IL-6 (*t*(159)=1.58; *p*=0.115). However, TNF-α was significantly lower in acAN-TP1 (log10 transformed M±SD = -0.20±0.13) compared with HC (-0.15±0.13) (*t*(159)=2.27; *p*=0.025). When removing recAN participants that were taking antidepressants (n=3), there remained no differences in BDNF (*t*(20)=-0.13; *p*=0.900), IL-6 (*t*(35)=0.68; *p*=0.501) or TNF-α (*t*(35)=0.76; *p*=0.453) compared with HC.

*SM 2.1.2. Cross-sectional differences in hippocampal (sub-)regions*

Table S1 displays the results of the between-group comparisons in hippocampal regions and subfields between acAN-TP1 and HC, whilst controlling for age, age^2^ and estimated total intracranial volume. Regions significant prior to FDR correction (n=27) are displayed in the main manuscript. After FDR correction, the right and left whole hippocampi were smaller in acAN-TP1, as well as the hippocampal head and tail bilaterally, the right hippocampal body and 16 subfields. In both hemispheres, the CA1 head, the CA4 head, the GC ML DG head, and the molecular layer of the hippocampus body and head were smaller in acAN-TP1. In the left hemisphere, the hippocampal-amygdalar transition area was smaller in the acAN-TP1 group. Additionally, in the right hemisphere, the CA1 body, CA3 head, GC ML DG body, and the presubiculum body and head were smaller in acAN-TP1.

Table S2 displays the results of the between-group comparisons in hippocampal region and subfield volumes between recAN and HC, also after controlling for age, age^2^ and estimated total intracranial volume. After FDR correction, no regions were significantly different between groups.

Table S1. Results from general linear models investigating between-group differences in hippocampal (sub-)regions between acAN-TP1 and HC, controlling for age, age^2^ and estimated total intracranial volume.

| Region | **Left hemisphere** | | | | | | |  | **Right hemisphere** | | | | | | |  |
| --- | --- | --- | --- | --- | --- | --- | --- | --- | --- | --- | --- | --- | --- | --- | --- | --- |
|  | B | SE | df | t | *p* | *q* | *d* |  | B | SE | df | t | *p* | *q* | *d* |  |
| Whole hippocampus | 70.03 | 19.12 | 159 | 3.66 | <0.001** | <0.001** | 0.58 |  | 98.76 | 19.87 | 159 | 4.97 | <0.001** | <0.001** | 0.79 |  |
| Whole hippocampal body | 12.19 | 7.14 | 159 | 1.71 | 0.090 | 0.122 | 0.27 |  | 24.36 | 7.64 | 159 | 3.19 | 0.002** | 0.006** | 0.51 |  |
| Whole hippocampal head | 35.47 | 11.09 | 159 | 3.20 | 0.002** | 0.006** | 0.51 |  | 50.90 | 11.00 | 159 | 4.64 | <0.001** | <0.001** | 0.74 |  |
| Hippocampal tail | 22.37 | 5.27 | 159 | 4.24 | <0.001** | <0.001** | 0.67 |  | 23.49 | 5.42 | 159 | 4.33 | <0.001** | <0.001** | 0.69 |  |
| CA1 body | 2.74 | 1.75 | 159 | 1.56 | 0.120 | 0.158 | 0.25 |  | 4.50 | 1.58 | 159 | 2.85 | 0.005** | 0.012* | 0.45 |  |
| CA1 head | 12.44 | 3.73 | 159 | 3.34 | 0.001** | 0.004** | 0.53 |  | 17.33 | 4.06 | 159 | 4.27 | <0.001** | <0.001** | 0.68 |  |
| CA3 body | -0.81 | 1.23 | 159 | -0.66 | 0.509 | 0.544 | 0.10 |  | 0.39 | 1.27 | 159 | 0.31 | 0.759 | 0.759 | 0.05 |  |
| CA3 head | 2.25 | 1.23 | 159 | 1.83 | 0.069 | 0.097 | 0.29 |  | 4.11 | 1.32 | 159 | 3.13 | 0.002** | 0.006** | 0.50 |  |
| CA4 body | 1.26 | 0.92 | 159 | 1.38 | 0.171 | 0.217 | 0.22 |  | 2.35 | 1.09 | 159 | 2.17 | 0.032* | 0.058 | 0.34 |  |
| CA4 head | 2.74 | 1.05 | 159 | 2.62 | 0.010* | 0.020* | 0.42 |  | 4.09 | 1.04 | 159 | 3.92 | <0.001** | <0.001** | 0.62 |  |
| Fimbria | -0.90 | 1.39 | 159 | -0.65 | 0.518 | 0.544 | 0.10 |  | 1.90 | 1.02 | 159 | 1.86 | 0.064 | 0.097 | 0.30 |  |
| GC ML DG body | 1.80 | 0.97 | 159 | 1.85 | 0.066 | 0.097 | 0.29 |  | 2.71 | 1.03 | 159 | 2.63 | 0.009** | 0.020* | 0.42 |  |
| GC ML DG head | 3.60 | 1.25 | 159 | 2.88 | 0.005** | 0.011* | 0.46 |  | 5.34 | 1.33 | 159 | 4.03 | <0.001** | <0.001** | 0.64 |  |
| HATA | 2.47 | 0.64 | 159 | 3.83 | <0.001** | <0.001** | 0.61 |  | 1.58 | 0.71 | 159 | 2.22 | 0.028* | 0.053 | 0.35 |  |
| Hippocampal fissure | -3.71 | 1.83 | 159 | -2.03 | 0.044* | 0.077 | 0.32 |  | -0.85 | 1.82 | 159 | -0.47 | 0.642 | 0.658 | 0.07 |  |
| Molecular layer HP body | 3.90 | 1.60 | 159 | 2.43 | 0.016* | 0.032* | 0.39 |  | 6.65 | 1.78 | 159 | 3.73 | <0.001** | <0.001** | 0.59 |  |
| Molecular layer HP head | 6.83 | 2.21 | 159 | 3.10 | 0.002** | 0.006** | 0.49 |  | 10.00 | 2.21 | 159 | 4.53 | <0.001** | <0.001** | 0.72 |  |
| Parasubiculum | 0.99 | 0.80 | 159 | 1.24 | 0.218 | 0.254 | 0.20 |  | 1.42 | 0.73 | 159 | 1.96 | 0.052 | 0.084 | 0.31 |  |
| Presubiculum body | 2.76 | 2.08 | 159 | 1.33 | 0.186 | 0.230 | 0.21 |  | 4.34 | 1.42 | 159 | 3.05 | 0.003** | 0.007** | 0.48 |  |
| Presubiculum head | 1.94 | 1.06 | 159 | 1.84 | 0.068 | 0.097 | 0.29 |  | 3.48 | 1.01 | 159 | 3.44 | <0.001** | <0.001** | 0.55 |  |
| Subiculum body | 1.45 | 1.89 | 159 | 0.77 | 0.445 | 0.492 | 0.12 |  | 1.53 | 1.70 | 159 | 0.90 | 0.369 | 0.419 | 0.14 |  |
| Subiculum head | 2.20 | 1.72 | 159 | 1.28 | 0.203 | 0.243 | 0.20 |  | 3.55 | 1.79 | 159 | 1.98 | 0.049* | 0.083 | 0.31 |  |

*Notes.* **significant at the *p*<0.01 threshold. *significant at the *p*<0.05 threshold. Abbreviations: B = Unstandardised Beta; CA = cornu ammonis; df = degrees of freedom; FDR = false discovery rate; GC ML DG = granule cell and molecular layer of the dentate gyrus; HATA = hippocampus-amygdala-transition-area; HP = hippocampus; SE = standard error.

Table S2. Results from general linear models investigating between-group differences in hippocampal (sub-)regions between recAN and HC, controlling for age, age^2^ and estimated total intracranial volume.

| Region | **Left hemisphere** | | | | | | |  | **Right hemisphere** | | | | | | |
| --- | --- | --- | --- | --- | --- | --- | --- | --- | --- | --- | --- | --- | --- | --- | --- |
|  | B | SE | df | t | *p* | *q* | *d* |  | B | SE | df | t | *p* | *q* | *d* |
| Whole hippocampus | -2.46 | 42.28 | 35 | -0.06 | 0.954 | 0.978 | 0.02 |  | 1.10 | 40.21 | 35 | 0.03 | 0.978 | 0.978 | <0.01 |
| Whole hippocampal body | -1.12 | 13.25 | 35 | -0.08 | 0.933 | 0.959 | 0.03 |  | -6.62 | 12.92 | 35 | -0.51 | 0.612 | 0.888 | 0.17 |
| Whole hippocampal head | 13.24 | 28.21 | 35 | 0.47 | 0.642 | 0.898 | 0.16 |  | 22.20 | 26.24 | 35 | 0.85 | 0.403 | 0.847 | 0.29 |
| Hippocampal tail | -14.58 | 11.21 | 35 | -1.30 | 0.202 | 0.720 | 0.44 |  | -14.48 | 12.22 | 35 | -1.19 | 0.244 | 0.720 | 0.40 |
| CA1 body | 3.56 | 3.45 | 35 | 1.03 | 0.309 | 0.720 | 0.35 |  | 2.74 | 2.58 | 35 | 1.06 | 0.295 | 0.720 | 0.36 |
| CA1 head | 7.08 | 9.39 | 35 | 0.75 | 0.456 | 0.865 | 0.25 |  | 11.98 | 9.31 | 35 | 1.29 | 0.207 | 0.720 | 0.44 |
| CA3 body | 2.86 | 2.44 | 35 | 1.17 | 0.249 | 0.720 | 0.40 |  | 0.67 | 2.25 | 35 | 0.30 | 0.768 | 0.943 | 0.10 |
| CA3 head | 0.22 | 2.73 | 35 | 0.08 | 0.936 | 0.959 | 0.03 |  | 3.20 | 2.75 | 35 | 1.16 | 0.253 | 0.720 | 0.39 |
| CA4 body | 2.07 | 1.66 | 35 | 1.25 | 0.219 | 0.720 | 0.42 |  | 0.88 | 1.73 | 35 | 0.51 | 0.613 | 0.888 | 0.17 |
| CA4 head | 2.26 | 2.51 | 35 | 0.90 | 0.373 | 0.825 | 0.30 |  | 3.97 | 2.21 | 35 | 1.80 | 0.081 | 0.565 | 0.61 |
| Fimbria | -5.17 | 2.27 | 35 | -2.28 | 0.029* | 0.479 | 0.77 |  | -0.68 | 1.83 | 35 | -0.37 | 0.713 | 0.943 | 0.13 |
| GC ML DG body | 1.28 | 1.76 | 35 | 0.72 | 0.474 | 0.865 | 0.24 |  | 0.30 | 1.60 | 35 | 0.19 | 0.853 | 0.943 | 0.06 |
| GC ML DG head | 2.22 | 3.01 | 35 | 0.74 | 0.466 | 0.865 | 0.25 |  | 4.80 | 2.81 | 35 | 1.71 | 0.097 | 0.581 | 0.58 |
| HATA | -0.85 | 1.553 | 35 | -0.54 | 0.589 | 0.888 | 0.18 |  | 0.46 | 1.47 | 35 | 0.31 | 0.756 | 0.943 | 0.11 |
| Hippocampal fissure | 0.89 | 4.620 | 35 | 0.19 | 0.849 | 0.943 | 0.06 |  | 2.49 | 4.14 | 35 | 0.60 | 0.551 | 0.888 | 0.20 |
| Molecular layer HP body | 0.93 | 3.1503 | 35 | 0.29 | 0.770 | 0.943 | 0.10 |  | 0.55 | 2.89 | 35 | 0.19 | 0.852 | 0.943 | 0.06 |
| Molecular layer HP head | 3.68 | 5.3671 | 35 | 0.69 | 0.498 | 0.871 | 0.23 |  | 5.49 | 5.04 | 35 | 1.09 | 0.283 | 0.720 | 0.37 |
| Parasubiculum | -1.99 | 1.5605 | 35 | -1.28 | 0.210 | 0.720 | 0.43 |  | -3.45 | 1.63 | 35 | -2.12 | 0.042* | 0.479 | 0.72 |
| Presubiculum body | -6.68 | 3.39125 | 35 | -1.97 | 0.057 | 0.479 | 0.67 |  | -5.31 | 2.47 | 35 | -2.15 | 0.039* | 0.479 | 0.73 |
| Presubiculum head | -0.26 | 2.277 | 35 | -0.11 | 0.910 | 0.959 | 0.04 |  | -2.45 | 2.32 | 35 | -1.06 | 0.296 | 0.720 | 0.36 |
| Subiculum body | 0.04 | 3.543 | 35 | 0.01 | 0.992 | 0.992 | <0.01 |  | -5.78 | 2.91 | 35 | -1.98 | 0.055 | 0.479 | 0.67 |
| Subiculum head | 0.88 | 3.3124 | 35 | 0.27 | 0.792 | 0.943 | 0.09 |  | -1.80 | 3.31 | 35 | -0.54 | 0.591 | 0.888 | 0.18 |

*Notes.* *significant at the *p*<0.05 threshold. Abbreviations: B = Unstandardised Beta; CA = cornu ammonis; df = degrees of freedom; FDR = false discovery rate; GC ML DG = granule cell and molecular layer of the dentate gyrus; HATA = hippocampus-amygdala-transition-area; HP = hippocampus; SE = standard error.

*SM 2.1.3. Main effects of molecule in general linear models investigating the association between TNF-α, IL-6 and BDNF and cross-sectional differences in hippocampal (sub-)regions*

The main effect of molecule in the general linear models investigating the association between TNF-α, IL-6 and BDNF and cross-sectional differences in hippocampal (sub-)regions between acAN-TP1 and HC, and recAN and HC, can be seen in Table S3 and S4 respectively. After FDR correction, the main effects of molecule were non-significant for all general linear models.

Table S3. Effect of molecule in general linear model investigating the association between TNF-α, IL-6 or BDNF on the volume of hippocampal regions (n=27) volumetrically different between acAN-TP1 and HC, after controlling for age, age^2^ and eTIV. Reported statistics refer to the molecule term.

| Region | **TNF-α** | | | | |  |  | **IL-6** | | | | |  |  | **BDNF** | | | | |  |
| --- | --- | --- | --- | --- | --- | --- | --- | --- | --- | --- | --- | --- | --- | --- | --- | --- | --- | --- | --- | --- |
|  | B | SE | t | *p* | *q* | *d* |  | B | SE | t | *p* | *q* | *d* |  | B | SE | t | *p* | *q* | *d* |
| **Left hemisphere** |  |  |  |  |  |  |  |  |  |  |  |  |  |  |  |  |  |  |  |  |
| Whole hippocampus | -250.81 | 140.54 | -1.79 | 0.076 | 0.206 | 0.28 |  | -57.00 | 46.63 | -1.22 | 0.223 | 0.332 | 0.20 |  | 34.95 | 171.66 | 0.20 | 0.839 | 0.895 | 0.04 |
| Hippocampal head | -96.38 | 82.11 | -1.17 | 0.242 | 0.398 | 0.19 |  | -40.84 | 26.97 | -1.51 | 0.132 | 0.235 | 0.24 |  | -10.26 | 102.28 | -0.10 | 0.920 | 0.950 | 0.02 |
| Hippocampal tail | -59.71 | 38.88 | -1.54 | 0.127 | 0.274 | 0.25 |  | 3.88 | 12.90 | 0.30 | 0.764 | 0.825 | 0.05 |  | 26.79 | 45.58 | 0.59 | 0.558 | 0.803 | 0.10 |
| CA1 head | -19.92 | 27.66 | -0.72 | 0.472 | 0.612 | 0.11 |  | -8.10 | 9.06 | -0.90 | 0.372 | 0.503 | 0.14 |  | -8.66 | 34.12 | -0.25 | 0.800 | 0.895 | 0.05 |
| CA4 head | -7.70 | 7.78 | -0.99 | 0.324 | 0.464 | 0.16 |  | -5.77 | 2.53 | -2.28 | 0.024* | 0.055 | 0.36 |  | 2.20 | 9.54 | 0.23 | 0.818 | 0.895 | 0.04 |
| GC ML DG head | -6.77 | 9.30 | -0.73 | 0.468 | 0.610 | 0.12 |  | -6.07 | 3.03 | -2.00 | 0.047* | 0.097 | 0.32 |  | 2.20 | 11.38 | 0.19 | 0.847 | 0.895 | 0.03 |
| Fissure | -13.88 | 13.54 | -1.03 | 0.307 | 0.450 | 0.16 |  | -6.80 | 4.42 | -1.54 | 0.126 | 0.228 | 0.25 |  | 4.56 | 15.73 | 0.29 | 0.772 | 0.885 | 0.05 |
| HATA | 6.02 | 4.74 | 1.27 | 0.206 | 0.357 | 0.20 |  | -0.89 | 1.58 | -0.56 | 0.576 | 0.700 | 0.09 |  | -2.14 | 5.57 | -0.38 | 0.702 | 0.867 | 0.07 |
| Molecular layer HP body | -16.96 | 11.87 | -1.43 | 0.155 | 0.308 | 0.23 |  | -3.49 | 3.93 | -0.89 | 0.376 | 0.503 | 0.14 |  | 9.63 | 13.91 | 0.69 | 0.490 | 0.776 | 0.12 |
| Molecular layer HP head | -25.57 | 16.25 | -1.57 | 0.118 | 0.265 | 0.25 |  | -6.88 | 5.37 | -1.28 | 0.202 | 0.308 | 0.20 |  | -4.25 | 20.38 | -0.21 | 0.835 | 0.895 | 0.04 |
| **Right hemisphere** |  |  |  |  |  |  |  |  |  |  |  |  |  |  |  |  |  |  |  |  |
| Whole hippocampus | -260.79 | 146.23 | -1.78 | 0.076 | 0.206 | 0.28 |  | -84.30 | 47.92 | -1.76 | 0.081 | 0.158 | 0.28 |  | 147.39 | 176.27 | 0.84 | 0.405 | 0.695 | 0.15 |
| Hippocampal body | -90.70 | 56.41 | -1.61 | 0.110 | 0.256 | 0.26 |  | -30.50 | 18.55 | -1.64 | 0.102 | 0.197 | 0.26 |  | 77.82 | 65.22 | 1.19 | 0.235 | 0.529 | 0.21 |
| Hippocampal head | -104.57 | 81.13 | -1.29 | 0.199 | 0.355 | 0.21 |  | -37.38 | 26.63 | -1.40 | 0.162 | 0.268 | 0.22 |  | 27.53 | 100.95 | 0.27 | 0.786 | 0.889 | 0.05 |
| Hippocampal tail | -65.52 | 40.05 | -1.64 | 0.104 | 0.255 | 0.26 |  | -16.42 | 13.13 | -1.25 | 0.213 | 0.322 | 0.20 |  | 42.04 | 47.33 | 0.89 | 0.376 | 0.658 | 0.16 |
| CA1 body | -7.58 | 11.75 | -0.65 | 0.520 | 0.651 | 0.10 |  | -8.47 | 3.80 | -2.23 | 0.027* | 0.061 | 0.36 |  | 8.91 | 14.14 | 0.63 | 0.530 | 0.791 | 0.11 |
| CA1 head | -34.40 | 30.11 | -1.14 | 0.255 | 0.409 | 0.18 |  | -14.18 | 9.83 | -1.44 | 0.151 | 0.263 | 0.23 |  | 13.63 | 37.19 | 0.37 | 0.715 | 0.867 | 0.07 |
| CA3 head | -5.34 | 9.78 | -0.55 | 0.586 | 0.711 | 0.09 |  | -6.64 | 3.19 | -2.08 | 0.039* | 0.084 | 0.33 |  | 12.14 | 11.93 | 1.02 | 0.311 | 0.570 | 0.18 |
| CA4 body | -14.80 | 8.01 | -1.85 | 0.066 | 0.185 | 0.30 |  | -5.30 | 2.63 | -2.01 | 0.046* | 0.097 | 0.32 |  | 12.36 | 9.29 | 1.33 | 0.186 | 0.468 | 0.24 |
| CA4 head | -8.38 | 7.75 | -1.08 | 0.281 | 0.432 | 0.17 |  | -5.43 | 2.53 | -2.15 | 0.033* | 0.073 | 0.34 |  | 10.57 | 9.60 | 1.10 | 0.273 | 0.570 | 0.20 |
| GC ML DG body | -12.95 | 7.61 | -1.70 | 0.091 | 0.235 | 0.27 |  | -5.22 | 2.50 | -2.09 | 0.038* | 0.083 | 0.33 |  | 13.36 | 8.75 | 1.53 | 0.129 | 0.381 | 0.27 |
| GC ML DG head | -7.84 | 9.85 | -0.80 | 0.427 | 0.578 | 0.13 |  | -6.43 | 3.22 | -2.00 | 0.047* | 0.097 | 0.32 |  | 12.85 | 12.18 | 1.05 | 0.294 | 0.570 | 0.19 |
| HATA | 8.20 | 5.26 | 1.56 | 0.121 | 0.269 | 0.25 |  | -1.98 | 1.74 | -1.13 | 0.259 | 0.368 | 0.18 |  | 4.66 | 6.45 | 0.72 | 0.471 | 0.774 | 0.13 |
| Molecular layer HP body | -15.62 | 13.22 | -1.18 | 0.239 | 0.397 | 0.19 |  | -8.15 | 4.30 | -1.89 | 0.060 | 0.122 | 0.30 |  | 19.00 | 15.35 | 1.24 | 0.218 | 0.515 | 0.22 |
| Molecular layer HP head | -26.46 | 16.25 | -1.63 | 0.106 | 0.256 | 0.26 |  | -6.48 | 5.36 | -1.21 | 0.229 | 0.335 | 0.19 |  | 3.53 | 20.28 | 0.17 | 0.862 | 0.895 | 0.03 |
| Presubiculum body | -14.49 | 10.51 | -1.38 | 0.170 | 0.327 | 0.22 |  | 4.75 | 3.47 | 1.37 | 0.173 | 0.273 | 0.22 |  | -4.08 | 12.65 | -0.32 | 0.748 | 0.867 | 0.06 |
| Presubiculum head | -11.05 | 7.44 | -1.49 | 0.139 | 0.280 | 0.24 |  | 0.77 | 2.47 | 0.31 | 0.755 | 0.823 | 0.05 |  | -8.63 | 8.89 | -0.97 | 0.334 | 0.595 | 0.17 |
| Subiculum head | -19.73 | 13.23 | -1.49 | 0.138 | 0.280 | 0.24 |  | 2.59 | 4.37 | 0.59 | 0.554 | 0.684 | 0.09 |  | -19.21 | 15.95 | -1.21 | 0.231 | 0.529 | 0.21 |

*Notes.* *significant at the *p*<0.05 threshold. Abbreviations: B = Unstandardised Beta; BDNF = brain-derived neurotrophic factor; CA = cornu ammonis; *d* = Cohen’s *d*; FDR = false discovery rate; GC ML DG = granule cell and molecular layer of the dentate gyrus; HATA = hippocampus-amygdala-transition-area; HP = hippocampus; IL-6 = interleukin-6; SE = standard error; TNF-α = tumor necrosis factor-alpha.

Table S4. Effect of molecule in general linear model investigating the association between TNF-α, IL-6 or BDNF on the volume of hippocampal regions (n=3) volumetrically different between recAN and HC, after controlling for age, age^2^ and eTIV. Reported statistics refer to the molecule term.

| Region | **TNF-α** | | | | | |  | **IL-6** | | | | | |  | **BDNF** | | | | | |
| --- | --- | --- | --- | --- | --- | --- | --- | --- | --- | --- | --- | --- | --- | --- | --- | --- | --- | --- | --- | --- |
|  | B | SE | t | *p* | *q* | *d* |  | B | SE | t | *p* | *q* | *d* |  | B | SE | t | *p* | *q* | *d* |
| **Left hemisphere** | | | | | | | | | | | | | | | | | | | | |
| Fimbria | -14.08 | 20.81 | -0.68 | 0.503 | 0.503 | 0.24 |  | -1.17 | 7.54 | -0.16 | 0.878 | 0.932 | 0.05 |  | -80.37 | 36.44 | -2.21 | 0.042* | 0.123 | 1.10 |
| **Right hemisphere** | | | | | | | | | | | | | | | | | | | | |
| Parasubiculum | -13.12 | 14.91 | -0.88 | 0.385 | 0.503 | 0.31 |  | -0.47 | 5.42 | -0.09 | 0.932 | 0.932 | 0.03 |  | 14.99 | 24.54 | 0.61 | 0.550 | 0.550 | 0.31 |
| Presubiculum body | -23.37 | 22.53 | -1.04 | 0.307 | 0.503 | 0.36 |  | -7.68 | 8.10 | -0.95 | 0.350 | 0.932 | 0.33 |  | -59.27 | 31.93 | -1.86 | 0.082 | 0.123 | 0.93 |

*Notes.* Abbreviations: B = Unstandardised Beta; BDNF = brain-derived neurotrophic factor; *d* = Cohen’s *d;* FDR = false discovery rate; IL-6 = interleukin-6; SE = standard error; TNF-α = tumor necrosis factor-alpha.

**SM 2.2. Longitudinal results**

*SM 2.2.1. Demographic and clinical characteristics excluding patients on antidepressant medication and with the binge-purge subtype of anorexia nervosa*

When removing patients on antidepressants (n=4 at TP2), BDNF increased from TP1 to TP2 (F(1,50)=4.78; *p*=0.033), and concentrations of IL-6 (F(1,63)=1.60; *p*=0.210) and TNF-α (F(1,59)=0.18; *p*=0.671) remained the same. However, when removing patients with the AN-BP subtype (n=11), concentrations of BDNF did not increase over time (F(1,50)=3.26; *p*=0.077), nor did IL-6 (F(1,45)=0.42; *p*=0.519) or TNF-α (F(1,49)=0.72; *p*=0.399).

*SM 2.2.2. Differences in hippocampal (sub-)regions between AN-TP1 and AN-TP2*

Table S5 displays the results of the longitudinal comparisons in hippocampal sub-regions between acAN-TP1 and acAN-TP2, whilst controlling for age, age^2^, estimated total intracranial volume and ΔBMI-SDS. Regions significant prior to FDR correction (n=16) are displayed in the main manuscript. After FDR correction, a total of 13 regions were significantly different between acAN-TP1 and acAN-TP2. Regions that were larger at acAN-TP2 in the left hemisphere include the whole hippocampus, hippocampal tail, GC ML DG head, molecular layer HP body and presubiculum body, whereas the CA3 body and fissure were smaller at TP2. In the right hemisphere, the whole hippocampus, hippocampal tail, hippocampal-amygdala transition area and presubiculum body were larger in acAN-TP2, whereas again the CA3 body and fissure were smaller.

*SM 2.2.3*. *Sensitivity analyses replicating the* *association between log10 ΔBDNF and longitudinal hippocampal changes over and above ΔBMI-SDS, after removing patients on antidepressant medication and with the binge-purge subtype of anorexia nervosa*

Table S6 shows the full results of the sensitivity analyses, of linear mixed effects models assessing the effects of changes in BDNF on longitudinal changes in the volumes of the left and right whole hippocampi between acAN-TP1 and acAN-TP2, after removing patients with the AN binge-purge subtype (n=11) or those on anti-depressant medication (n=4). All results from all sensitivity analyses remained significant apart from one, whereby the effect of ΔBDNF on the right whole hippocampus was no longer significant after removing participants with the AN-BP subtype.

Table S5. Results from linear mixed effects models investigating longitudinal differences in hippocampal (sub-)regions between TP1 and TP2 in patients with acAN, controlling for age, age^2^, estimated total intracranial volume and ΔBMI-SDS.

| Region | **Left hemisphere** | | | | | |  |  | **Right hemisphere** | | | | | |  |
| --- | --- | --- | --- | --- | --- | --- | --- | --- | --- | --- | --- | --- | --- | --- | --- |
|  | B | SE | Df | t | *p* | *q* | *d* |  | B | SE | Df | t | *p* | *q* | *d* |
| Whole hippocampus | 36.07 | 10.07 | 63.24 | 3.58 | 0.002** | 0.004** | 0.14 |  | 29.19 | 8.47 | 65.74 | 3.45 | 0.004** | 0.004** | 0.11 |
| Whole hippocampal body | 8.42 | 4.24 | 62.72 | 1.99 | 0.162 | 0.357 | 0.09 |  | 3.04 | 3.87 | 64.05 | 0.78 | 0.832 | 0.970 | 0.03 |
| Whole hippocampal head | 12.99 | 4.89 | 65.33 | 2.66 | 0.034* | 0.103 | 0.08 |  | 6.21 | 4.54 | 65.48 | 1.37 | 0.464 | 0.690 | 0.04 |
| Hippocampal tail | 14.80 | 3.99 | 59.91 | 3.71 | 0.002** | 0.011* | 0.22 |  | 20.23 | 3.52 | 62.08 | 5.75 | <0.001*** | <0.001*** | 0.27 |
| CA1 body | 0.52 | 0.80 | 62.70 | 0.65 | 0.895 | 0.998 | 0.03 |  | -1.48 | 0.62 | 66.09 | -2.39 | 0.067 | 0.187 | -0.07 |
| CA1 head | 2.00 | 1.77 | 65.23 | 1.13 | 0.621 | 0.790 | 0.04 |  | 2.60 | 1.63 | 66.73 | 1.60 | 0.328 | 0.552 | 0.05 |
| CA3 body | -2.24 | 0.73 | 64.89 | -3.06 | 0.011* | 0.043* | -0.10 |  | -2.25 | 0.66 | 65.95 | -3.40 | 0.004** | 0.022* | -0.10 |
| CA3 head | 0.75 | 0.66 | 62.43 | 1.14 | 0.611 | 0.790 | 0.05 |  | -1.13 | 0.60 | 63.85 | -1.87 | 0.202 | 0.405 | -0.06 |
| CA4 body | -0.11 | 0.67 | 61.10 | -0.16 | 0.998 | 1.000 | -0.01 |  | -0.68 | 0.72 | 63.00 | -0.94 | 0.740 | 0.889 | -0.04 |
| CA4 head | 1.43 | 0.65 | 60.72 | 2.20 | 0.103 | 0.255 | 0.10 |  | -0.92 | 0.53 | 63.11 | -1.76 | 0.251 | 0.458 | -0.06 |
| Fimbria | 0.95 | 0.71 | 62.39 | 1.35 | 0.476 | 0.690 | 0.08 |  | 1.13 | 0.75 | 59.46 | 1.51 | 0.376 | 0.607 | 0.11 |
| GC ML DG body | 1.52 | 0.65 | 61.43 | 2.32 | 0.080 | 0.209 | 0.12 |  | 0.09 | 0.74 | 62.58 | 0.12 | 0.999 | 1.000 | 0.01 |
| GC ML DG head | 2.51 | 0.68 | 61.76 | 3.68 | 0.002** | 0.011* | 0.16 |  | 0.03 | 0.54 | 65.08 | 0.05 | 1.000 | 1.000 | 0.00 |
| HATA | 0.87 | 0.54 | 59.28 | 1.61 | 0.320 | 0.552 | 0.10 |  | 1.43 | 0.43 | 60.32 | 3.34 | 0.005** | 0.024* | 0.16 |
| Hippocampal fissure | -13.96 | 2.37 | 58.14 | -5.90 | <0.001*** | <0.001*** | -0.49 |  | -14.65 | 2.44 | 59.09 | -6.01 | <0.001*** | <0.001*** | -0.52 |
| Molecular layer HP body | 4.34 | 1.13 | 62.69 | 3.83 | 0.001** | 0.009** | 0.19 |  | 2.79 | 1.01 | 64.03 | 2.77 | 0.026* | 0.090 | 0.11 |
| Molecular layer HP head | 2.99 | 1.12 | 63.60 | 2.67 | 0.034* | 0.103 | 0.10 |  | 1.30 | 1.07 | 63.60 | 1.22 | 0.561 | 0.760 | 0.04 |
| Parasubiculum | 0.60 | 0.41 | 61.98 | 1.49 | 0.390 | 0.607 | 0.07 |  | 0.74 | 0.38 | 61.24 | 1.92 | 0.185 | 0.389 | 0.08 |
| Presubiculum body | 3.85 | 1.17 | 62.85 | 3.28 | 0.006** | 0.026* | 0.14 |  | 5.18 | 1.09 | 59.66 | 4.77 | <0.001*** | <0.001*** | 0.27 |
| Presubiculum head | 1.37 | 0.77 | 62.27 | 1.78 | 0.242 | 0.458 | 0.09 |  | 1.36 | 0.63 | 62.75 | 2.17 | 0.109 | 0.255 | 0.09 |
| Subiculum body | -0.48 | 0.93 | 64.47 | -0.52 | 0.944 | 1.000 | -0.02 |  | -1.67 | 1.32 | 63.48 | -1.26 | 0.530 | 0.742 | -0.07 |
| Subiculum head | 0.58 | 0.91 | 63.82 | 0.63 | 0.903 | 0.998 | 0.02 |  | 0.89 | 0.91 | 63.19 | 0.98 | 0.719 | 0.888 | 0.04 |

*Notes.* **significant at the *p*<0.01 threshold. *significant at the *p*<0.05 threshold. Abbreviations: B = Unstandardised Beta; CA = cornu ammonis; *d* = Cohen’s *d*; df = degrees of freedom; FDR = false discovery rate; GC ML DG = granule cell and molecular layer of the dentate gyrus; HATA = hippocampus-amygdala-transition-area; HP = hippocampus; SE = standard error.

Table S6. The contribution of ΔBDNF to longitudinal differences in hippocampal (sub-)regions between TP1 and TP2 in patients with acAN after controlling for age, age^2^, estimated total intracranial volume and ΔBMI-SDS, excluding patients with the AN binge-purge subtype (n=11) and those currently using anti-depressant medication (n=4).

| Region | **Excluding AN-BP subtype** | | | | |  |  |  | | **Excluding medication usage** | | | | | |
| --- | --- | --- | --- | --- | --- | --- | --- | --- | --- | --- | --- | --- | --- | --- | --- |
|  | B | SE | t | *p* | *q* | *d* |  | B | SE | | t | *p* | *q* | *d* |  |
| **Left hemisphere** |  |  |  |  |  |  |  |  |  | |  |  |  |  |  |
| Whole hippocampus | 311.61 | 120.95 | 2.58 | 0.014* | 0.028* | 0.37 |  | 331.25 | 120.69 | | 2.74 | 0.009** | 0.018* | 0.38 |  |
| **Right hemisphere** |  |  |  |  |  |  |  |  |  | |  |  |  |  |  |
| Whole hippocampus | 183.84 | 91.45 | 2.01 | 0.052 | 0.052 | 0.29 |  | 183.05 | 86.71 | | 2.11 | 0.041* | 0.041* | 0.29 |  |

*Notes.* **significant at the *p*<0.01 threshold. *significant at the *p*<0.05 threshold. Abbreviations: B = Unstandardised Beta; AN-BP = anorexia nervosa binge-purge subtype; FDR = false discovery rate; SE = standard error.

**SM 2.3. Exploratory correlations**

Table S7. Pearson’s correlations investigating the association between BDNF and IL-6/ TNF-α in the cross-sectional (whole sample, AN and HC individually) and longitudinal samples.

|  | (Δ)BDNF | | |  | |
| --- | --- | --- | --- | --- | --- |
|  | *r* | df | *p* | *q* | |
| *Whole sample (cross-sectional)* | | | |  |  |
| IL-6 | 0.09 | 132 | 0.285 | 0.285 | |
| TNF-α | 0.15 | 132 | 0.080 | 0.160 | |
| *Controls (cross-sectional)* | | | |  |  |
| IL-6 | 0.17 | 66 | 0.162 | 0.162 | |
| TNF-α | 0.20 | 66 | 0.107 | 0.162 | |
| *AN (cross-sectional)* | | | |  |  |
| IL-6 | -0.02 | 64 | 0.897 | 0.897 | |
| TNF-α | 0.10 | 64 | 0.430 | 0.860 | |
| *AN (longitudinal)* | | | |  |  |
| ΔIL-6 | 0.24 | 41 | 0.118 | 0.118 | |
| ΔTNF-α | 0.28 | 41 | 0.070 | 0.118 | |

*Notes.* *significant at the *p*<0.05 threshold. Abbreviations: AN = anorexia nervosa; BDNF = brain-derived neurotrophic factor; df = degrees of freedom; IL-6 = interleukin-6; *r* = Spearman’s rho; TNF-α = tumor necrosis factor-alpha.

**References**

Bahnsen, K., Bernardoni, F., King, J. A., Geisler, D., Weidner, K., Roessner, V., . . . Ehrlich, S. (2022). Dynamic Structural Brain Changes in Anorexia Nervosa: A Replication Study, Mega-analysis, and Virtual Histology Approach. *Journal of the American Academy of Child & Adolescent Psychiatry*.

Bernardoni, F., King, J. A., Geisler, D., Stein, E., Jaite, C., Nätsch, D., . . . Roessner, V. (2016). Weight restoration therapy rapidly reverses cortical thinning in anorexia nervosa: A longitudinal study. *Neuroimage, 130*, 214-222.

Desikan, R. S., Ségonne, F., Fischl, B., Quinn, B. T., Dickerson, B. C., Blacker, D., . . . Hyman, B. T. (2006). An automated labeling system for subdividing the human cerebral cortex on MRI scans into gyral based regions of interest. *Neuroimage, 31*(3), 968-980.

Fichter, M., & Quadflieg, N. (2001). The structured interview for anorexic and bulimic disorders for DSM-IV and ICD-10 (SIAB-EX): reliability and validity. *European Psychiatry, 16*(1), 38-48.

Fichter, M. M., & Quadflieg, N. (1999). *Strukturiertes Inventar für anorektische und bulimische Essstörungen nach DSM-IV und ICD-10 (SIAB)*: Hogrefe.

Fischl, B. (2012). FreeSurfer. *Neuroimage, 62*(2), 774-781.

Fischl, B., Salat, D. H., Busa, E., Albert, M., Dieterich, M., Haselgrove, C., . . . Klaveness, S. (2002). Whole brain segmentation: automated labeling of neuroanatomical structures in the human brain. *Neuron, 33*(3), 341-355.

Iglesias, J. E., Van Leemput, K., Augustinack, J., Insausti, R., Fischl, B., Reuter, M., & Initiative, A. s. D. N. (2016). Bayesian longitudinal segmentation of hippocampal substructures in brain MRI using subject-specific atlases. *Neuroimage, 141*, 542-555.

Kong, X. Z., Postema, M. C., Guadalupe, T., de Kovel, C., Boedhoe, P. S., Hoogman, M., . . . Glahn, D. C. (2022). Mapping brain asymmetry in health and disease through the ENIGMA consortium. *Human brain mapping, 43*(1), 167-181.

Reuter, M., Schmansky, N. J., Rosas, H. D., & Fischl, B. (2012). Within-subject template estimation for unbiased longitudinal image analysis. *Neuroimage, 61*(4), 1402-1418.

Sämann, P. G., Iglesias, J. E., Gutman, B., Grotegerd, D., Leenings, R., Flint, C., . . . van Erp, T. G. (2022). FreeSurfer‐based segmentation of hippocampal subfields: A review of methods and applications, with a novel quality control procedure for ENIGMA studies and other collaborative efforts. *Human brain mapping, 43*(1), 207-233.

Saygin, Z. M., Osher, D. E., Augustinack, J., Fischl, B., & Gabrieli, J. D. (2011). Connectivity-based segmentation of human amygdala nuclei using probabilistic tractography. *Neuroimage, 56*(3), 1353-1361.

Steinhäuser, J. L., King, J. A., Tam, F. I., Seidel, M., Biemann, R., Wronski, M.-L., . . . Ehrlich, S. (2021). Is serum BDNF altered in acute, short-and long-term recovered restrictive type anorexia nervosa? *Nutrients, 13*(2), 432.

Steinhäuser, J. L., Wronski, M.-L., Keeler, J. L., Ehrlich, S., & King, J. A. (2022). Barking up the wrong biomarker? Correspondence to Shobeiri et al.(2022)“Serum and plasma levels of brain-derived neurotrophic factor in individuals with eating disorders (EDs): a systematic review and meta-analysis”. *Journal of Eating Disorders, 10*(1), 1-4.
